# *Xenopus tropicalis* Genome Re-Scaffolding and Re-Annotation Reach the Resolution Required for *In Vivo* ChIA-PET Analysis

**DOI:** 10.1371/journal.pone.0137526

**Published:** 2015-09-08

**Authors:** Nicolas Buisine, Xiaoan Ruan, Patrice Bilesimo, Alexis Grimaldi, Gladys Alfama, Pramila Ariyaratne, Fabianus Mulawadi, Jieqi Chen, Wing-Kin Sung, Edison T. Liu, Barbara A. Demeneix, Yijun Ruan, Laurent M. Sachs

**Affiliations:** 1 UMR CNRS 7221, Muséum National d'Histoire Naturelle, Paris, France; 2 The Jackson Laboratory of Genomic Medicine, Farmington, Connecticut, United States of America; 3 Department of Genetics and Developmental Biology, University of Connecticut, Farmington, Connecticut, United States of America; 4 Genome Institute of Singapore, Singapore, Singapore; 5 Watchfrog S.A.S., Evry, France; University of California, Los Angeles, UNITED STATES

## Abstract

Genome-wide functional analyses require high-resolution genome assembly and annotation. We applied ChIA-PET to analyze gene regulatory networks, including 3D chromosome interactions, underlying thyroid hormone (TH) signaling in the frog *Xenopus tropicalis*. As the available versions of *Xenopus tropicalis* assembly and annotation lacked the resolution required for ChIA-PET we improve the genome assembly version 4.1 and annotations using data derived from the paired end tag (PET) sequencing technologies and approaches (e.g., DNA-PET [gPET], RNA-PET etc.). The large insert (~10Kb, ~17Kb) paired end DNA-PET with high throughput NGS sequencing not only significantly improved genome assembly quality, but also strongly reduced genome “fragmentation”, reducing total scaffold numbers by ~60%. Next, RNA-PET technology, designed and developed for the detection of full-length transcripts and fusion mRNA in whole transcriptome studies (ENCODE consortia), was applied to capture the 5' and 3' ends of transcripts. These amendments in assembly and annotation were essential prerequisites for the ChIA-PET analysis of TH transcription regulation. Their application revealed complex regulatory configurations of target genes and the structures of the regulatory networks underlying physiological responses. Our work allowed us to improve the quality of *Xenopus tropicalis* genomic resources, reaching the standard required for ChIA-PET analysis of transcriptional networks. We consider that the workflow proposed offers useful conceptual and methodological guidance and can readily be applied to other non-conventional models that have low-resolution genome data.

## Introduction

The rapid advances and reduced costs of sequencing are increasing the number of genomes available for study [1]. However, functional post-genomic analyses require high quality genome assembly and annotation, and such levels have only been achieved for a few reference genomes including human, mouse, drosophila and yeast. Initial genome assembly and annotation is labor-intensive, involves sustained computation and manual curation efforts that will most often be incomplete. Regular updates and continued curation are required to maintain high standards in reference genome quality in current public databases (e.g. the ENCODE Project Consortium, [2,3,4,5], the modENCODE Consortium). In contrast, most newly completed genome projects undergoing *de novo* assembly produce draft genome assemblies and annotations with tens, or even hundreds of thousands of fragmented scaffolds, numerous gaps and sequencing errors (see for example [6,7]). Although these assemblies, together with genome annotation are valuable resources and provide a reasonable estimation of the gene repertoire, they carry little information on the medium/large scale structure of the genome. Too often the quality of draft genomes is a major limitation when trying to exploit maximally the flood of data produced by the recent advances in functional genomics. Alternative strategies and approaches are needed to improve assembly and annotation (see [8], [9] and [10] for a few examples). Current advances in high-throughput sequencing of genomic DNA and RNAs offer complementary technologies to re-annotate genomes and to use these high-resolution tools to shed new light on our perception of physiological and biological processes in non-conventional models.

In all chordates, post-embryonic development involves a complex set of molecular, cellular and anatomical transitions orchestrated by thyroid hormones (THs) signaling [11,12]. Anatomical changes are often dramatic, as it is well illustrated in flatfish and amphibian metamorphosis. In precocial birds, such as chicks, this transition corresponds to the hatching period. In mammals it is often equated to the perinatal period [13], for which the transition phenotype is anatomically subtler, but the changes in respiration, nutrition and sensory nervous system maturation are well marked. THs trigger complex transcriptional programs of cellular remodeling, affecting cell fate by inducing a variety of responses in different tissues/cell types, for instance apoptosis versus proliferation and tissue growth. These programs often occur simultaneously in different cells within a given organ or tissue. Thus, because of these cell specific responses, it is difficult to design experimental approaches to characterize the transcriptional programs underlying these responses, particularly in *in vivo* settings. To tackle this challenge, we use metamorphosis in *Xenopus (Silurana) tropicalis* (*X*. *tropicalis*) as a working model. Amphibian metamorphosis is the most contrasted of the TH-dependent post-embryonic transitions. During Anuran (frogs and toads) metamorphosis, an aquatic tadpole changes to an air-breathing frog, with almost all tissues undergoing profound remodeling [14,15]. A key feature of amphibian metamorphosis is that external organs can display opposite fates: the tadpole tail regresses and ultimately disappears whilst limbs grow *de novo* from limb buds. Amphibian metamorphosis is thus an attractive model to study the impacts of THs on transcriptional reprograming during post-embryonic development in vertebrates. As in other organisms, THs signaling is mediated by nuclear receptors (TRs) that modulate the transcriptional state of target genes [16]. Our overall aim is to determine the repertoire of TR mediated direct and indirect TH target genes and the characterization of TR-based regulatory networks.

Deriving the full repertoire of a transcription factor binding sites (TFBS), such as those recognized by TRs, can usually be achieved with a target-specific ChIP-Seq experiment, in which the DNA sequences bound to an affinity-purified transcription factor are sequenced [17]. The problem of genome wide profile maps is that TFBS are often located at great distances (>200Kb) from their cognate gene, and can even be located within another gene. Indeed, enhancers located far from their target genes functionally and physically interact with the transcriptional machinery at the target gene through DNA looping [18]. As a result, it is misleading to relate each TFBS to a given target gene solely using genomic proximity. In addition, TFBS can also be arranged into *cis* regulatory modules controlling the transcriptional status of several target genes at once, which may further complicate the assignment of a TFBS to its target genes [19]. In order to circumvent these problems, we used the ChIA-PET technology to identify TR binding sites across the whole genome in a manner similar to ChIP-Seq, but together with their physical interactions with distant regulatory regions [19,20]. To date, this approach is the only one providing direct evidence for the unambiguous identification of the gene sets directly regulated by transcription factors. Crucially, successful ChIA-PET analysis relies on high quality genome assembly and annotations, as “fragmented” assemblies lower the resolution of medium/large-scale (a few Mb) interaction maps. In addition, poor gene annotation, especially at 5' ends, would tend to associate transcriptional regulators to regions located outside of genes and would make ChIA-PET analysis pointless. Unfortunately, during the initial course of our ChIA-PET analysis of the TR transcriptional network during *X*. *tropicalis* metamorphosis, it became clear that the state of the genome assembly and annotation was the bottleneck of the analysis, as their resolution was too low to resolve accurate ChIA-PET locus assignment.

Here, we describe the technological framework used to improve annotation and re-scaffold the genome assembly of *X*. *tropicalis*, to reach the standard required for ChIA-PET analysis (large scaffold size, accurate annotation of gene transcriptional start sites). This genome is also an ideal test case because the genome size (~1.5Gb) is smaller than mammalian genomes, and the draft assembly, although “fragmented” ([21], ~20,000 scaffolds) is much less so than others (e.g. fish *Platipus lavaretus* with ~350,000 scaffolds [22], or, Panda *Ailuropoda melanoleuca* with >500,000 scaffolds, [23]). We used two PET-based technologies: large insert library with DNA-PET and full-length transcripts with RNA-PET (in a combination with paired end RNA-Seq), using NGS sequencing. This combined approach allows accurate capture of 5' and 3' transcript ends and variants, in a length-independent manner. The methods are remarkably powerful for improving gene annotation, permitting detection of transcripts and unambiguously identifying gene boundaries, alternative transcripts, transcript start sites (TSS) and termination sites (TTS) etc. We also used the connectivity information provided by the large insert DNA-PET to bridge scaffolds in the published assembly. This combined effort significantly reduced the total scaffolds in the genome assembly, corrected and improved the published version and set it to a quality level suitable for ChIA-PET analysis.

## Results

### Large insert DNA-PET significantly reduces the complexity of genome assembly

**Fig 1:**
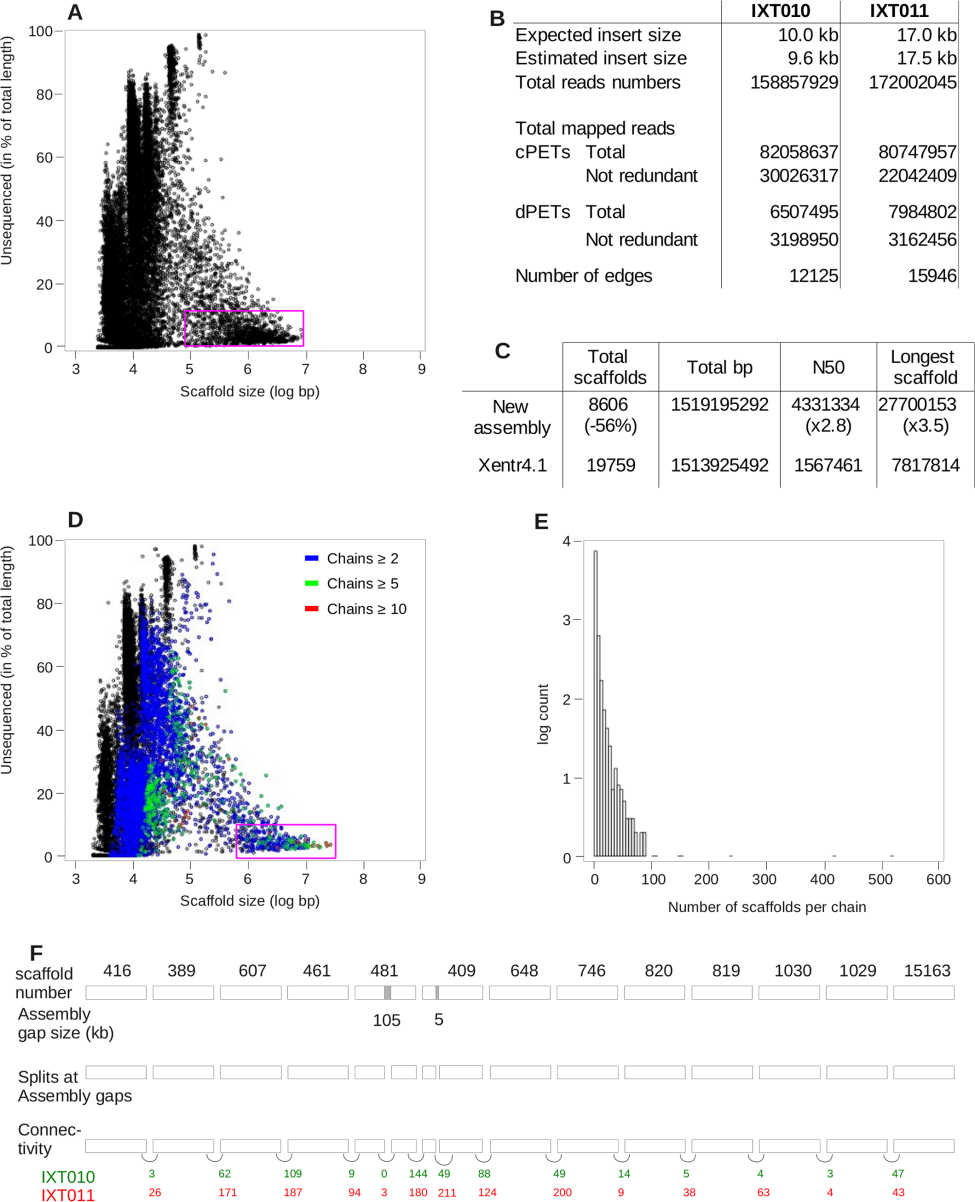
DNA-PET significantly improves genome assembly. A, D. Fraction of each scaffold left un-sequenced (expressed in per cent) as a function of size, in log scale. The initial genome assemble is plotted on the top, and the improved assembly of the bottom. The number of scaffolds chained together by dPETs ('chain length, in number of scaffolds) is indicated by blue, green and red colors. Purple circles denote the scaffolds corresponding to ~80% of the assembly. B. Raw DNA-PET data mapping statistics. C. Statistics of genome assembly improvement. E. Distribution of the number of scaffolds connected together with dPETs, showing that a large fraction of the total scaffolds are connected together into long chains. F. Example of re-scaffolding. A total of 15 scaffolds are linked together by dPETs. Tracks from top to bottom: scaffold name, assembly gap size, connectivity and number of dPETs per link for each DNA-PET library. The two scaffolds containing assembly gaps were split before re-scaffolding. Colored numbers indicate the number of independent dPETs supporting each connection, for each library.

Our starting point was the published *X*. *tropicalis* genome assembly (version 4.1). Although a new version was released during the course of this project, we elected not to use it because it suffers from several drawbacks (see Discussion). The version 4.1 assembly is composed of 19,759 scaffolds, with the top ~2000 scaffolds (longer than 20Kb) covering 80% of the assembly (~1.5Gb) [21]. The average scaffold size is about 76Kb (computed from [21] and [24]), which, given the average gene length (~28Kb), would be too small if one wants to probe the physical interactions connecting genes and distant regulatory elements by ChIA-PET. Typical long-range interactions often span of the order of 100Kb [25]. The vast majority of these scaffolds are not fully sequenced, leaving numerous, often large, assembly gaps (Fig 1A). The scaffolds can be classified into three main groups: 1) numerous small "scaffolds", composing ~30% of the total scaffolds, ~1.4% of the total sequence, actually have the size of Sanger sequencing reads or short contigs (up to 5Kb); 2) a collection of relative larger scaffolds (15Kb to 50Kb), with an average size consistent with that obtained from cosmid clones. Given that they contain large assembly gaps, they are more likely derived from un-assembled end-sequenced cosmid inserts. Finally, 3) the largest collection of scaffolds (>100Kb in size), that are most likely assembled from BAC clone sequences. The quality of this assembly is very similar to that of other draft assemblies (Supplementary data in [24]). Of note, among the 183,010 assembly gaps present in the published sequence, 84,126 (45.9%) are exactly 50bp long, which probably reflects some technical issues during the assembly process. Such systematic bias suggests that the real size of these "50bp gaps" is unknown and was set by default at this value. Unfortunately, this undocumented feature is an important source of confusion for people using these resources, who are unaware of its limitations and resolution.

We used DNA-PET technology to improve version 4.1 assembly. This technology was initially developed and applied to cancer research [26] and more recently to genome assembly [27,28]. The method involves sequencing of the two ends of size-defined large (e.g., 10Kb or 1Kb) DNA fragments, which are then mapped to the reference genome (S1 Fig). This provides important connectivity information over large genomic regions, which is often critical for genome assembly. The sequenced reads can be classified in two kinds of PETs: "concordant" PETs (cPETs), for which the two tags are mapped onto the same scaffold and at the given distance corresponding to the expected insert size. PETs, which fail to be classified as “concordants”, are labeled "discordant" PETs (dPETs). Therefore, the set of dPETs is composed of a mixture of PETs: those mapping on the same scaffold, but with discrepancies with respect to their insert size, and PETs where both tags map on different scaffolds. cPETs can be used to assess the quality of the datasets, estimate the number of assembly errors, the real size of assembled gaps of unknown length ("50bp gaps", see below) and dPETs mapping on the same scaffold to assess possible structural variations. dPETs mapping on different scaffolds can be used to improve the scaffolding by ordering un-assembled scaffolds with respect to each other. Two large insert DNA-PET libraries (10Kb and 17Kb) were created using kidney and liver genomic DNA. A summary of the DNA-PET statistical data is shown in Fig 1B. A total of 77.8 million uniquely mapped PETs were obtained for further mapping and downstream analysis.

The average insert size (mapped distance between two tags), estimated by measuring the mean genome span of cPETs, was 9.6Kb and 17.5Kb for the two libraries IXT010 and IXT011, respectively. This agrees well with the expected size (S2A Fig) and validates library quality. As expected, concordant PETs compose the vast majority of the dataset and dPETs accounted for 8 to 10% of the total PETs (Fig 1B, S1 Table), consistent with what is usually found in most DNA-PET datasets (for example see Fig 1C of [29], or Table 1 of [27]). In addition, genome-wide cPET coverage profiles were computed from each library by counting the number of cPETs in sliding 20Kb windows. They are highly correlated (*r* = 0.97, S2B Fig), thus showing the high quality and reproducibility of the datasets.

We first used the cPET datasets to estimate the size of the numerous "50bp gaps". We selected cPETs spanning a single gap of exactly 50bp and computed the average difference between the expected (9.6 or 17.5Kb) and the observed genome span of individual PETs. Positive values of this estimate indicate that the minimal region delineated by the cPETs is actually much longer than expected, presumably (but not only) because of the uncertainty attributed to the gap length. Negative values, meaning that the minimal region is actually shorter than expected, may result from structural polymorphism between the individual frogs we sequenced and the reference genome sequence. We found that more than half (46,249) of the "50bp gaps" out of (84,126) have an actual average size of ~500bp (S3 Fig). This increases the total size of the assembly by an extra 12.7Mb. In a typical case, the genome span of cPETs follows a bell shape distribution (see S4 Fig for a few illustrative examples). Interestingly, we also spotted a number of cases (~8%) where the genome span distribution of cPETs was bimodal, of which one, or both gap length estimates deviated significantly from expectation (S5 Fig). A similar procedure was used for assembly gaps of other sizes, of which a few examples of mono and bi-modal distributions are shown (S6 and S7 Figs). Altogether, the analysis increased the assembly size by ~21.8Mb in total. Simultaneously, we re-calculated the size of assembly gaps, since this can have a significant impact in estimates of the genomic distances between structural and/or functional elements. This information is important because 50bp long features can be difficult to see on genome browsers at scales of several Kb to hundreds of Kb, and their small apparent size can be misleadingly interpreted as a small assembly gap. Providing a real estimate of assembly gap size is a clear improvement and resolves a source of confusion.

In a number of cases, the cPET coverage drops to zero, meaning that the left and right hand sides of the breakpoint are not connected together at the resolution of the average DNA-PET insert size (~10Kb and ~17Kb). These assembly breakpoints can result either from structural polymorphisms between reference and sampled genomic DNA, or from various *scenarii* of mis-assemblies (*e*.*g*. the left and right hand sides of the breakpoints are each connected to a different scaffold, or the “50bp assembly gap” actually correspond to a region much longer than ~20Kb). Two examples are illustrated in S8 Fig. In both cases, cPET coverage drops to zero at a 50bp assembly gap. Note that by definition, the length of these 50bp gaps cannot be estimated based on the average cPET span. Interestingly, the breakpoints are flanked by one or two clusters of discordant PETs pointing to different scaffolds, strongly suggesting that based on our sequenced DNA sample, the left and right sides of the breakpoints are each connected to a different scaffold. Overall, we identified a total of 352 cPET coverage breakpoints, of which, 211 were re-connected to other scaffolds during the re-scaffolding process (see below).

We then used the dPETs mapping of different scaffolds to improve the physical contiguity of the scaffolds and to better describe the medium and large-scale structure of the *X*. *tropicalis* genome. From here onwards, the term 'dPET' will specifically refer to dPETs mapping on two different scaffolds. As a first step, and given the number of large assembly gaps that are susceptible to accommodate smaller scaffolds, scaffolds were split at gaps larger than 10Kb (corresponding to the smallest insert size of the DNA-PET libraries). This corresponded to 1,031 locations. We also split scaffolds at cPETs coverage breakpoints (see above). The two datasets were then combined and processed with the PE-Assembler software [30], which was previously shown to perform well (see methods, [31]). The statistics of the improved assembly are shown in Fig 1C. The total number of scaffolds is reduced by 56%, the N50 and the size of the largest scaffold increases by ~3 folds (2.8 x and 3.5 x, respectively). A total of 4,901 short contigs (~2–3Kb "scaffolds") could not be connected to other scaffolds. These originate from small contigs that are too short to be assembled given the insert size of our DNA-PET libraries (>9.6Kb). This means that two contiguous short scaffolds may not be directly connected by dPETs and would fail to assemble into longer chains. The remaining scaffolds could be associated into longer chains, of which 1,283 are composed of 5 scaffolds or more.

In order to visualize the improvements, we plotted the relationship between its size in bp (in log scale) and the percent of its sequence remaining to be determined (Fig 1D) for each scaffold and compared the result with the same representation of the published assembly (Fig 1A). Scaffolds located in the uppermost part of the plot correspond to scaffolds containing numerous or large assembly gaps. In contrast, scaffolds located at the lowermost part of the plot contain few, if any. Ideally, assembled scaffolds sequenced to completion, and ultimately chromosomes, should be located at the lower right side of the plot. Re-scaffolding with DNA-PET clearly shifts the distribution of the scaffolds corresponding to 80% of the sequence assembly (pink rectangle, Fig 1A) from 1Mb to towards to right hand side of the plot (Fig 1D).

Re-scaffolding with dPETs connected 26,703 scaffolds into 3,594 "chains" of various length, of which the frequency distribution is shown Fig 1E. The longest chain is composed of 519 scaffolds with an average chain length of 3.14 (median = 1). Although this may suggest that the re-scaffolding efficiency is rather low, finally, only a minority of scaffolds (4,901, ~18%) were not linked to another scaffold. When discarding these short "scaffolds", the average chain length rose to 5.12 (median = 3). In addition, smaller scaffolds filled 835 assembly gaps. An example of re-scaffolding is shown Fig 1F. The two libraries, built with different insert sizes, provided similar results, although the PET count of the edges originating from the 17Kb library tends to be higher than that of the 9.6Kb library. Two scaffolds containing large assembly gaps (105Kb in scaffolds_481 and 5Kb in scaffold_409), which were split in two before re-scaffolding, were effectively re-connected. Overall, the estimated link size between two scaffolds varies from < 1 to ~10Kb. In a number of cases, with link size < 6Kb, we could validate the link by conventional long-range PCR (data not shown).

Connectivity between scaffolds can be represented by direct acyclic graphs (S9–S11 Figs), where vertices correspond to scaffolds (drawn as ovals) and dPET links to edges. In these supporting information figures, vertices are labeled with the scaffold name, size and its original position in the scaffold, before the splitting at assembly gaps and assembly errors. With this representation, bubble-like structures correspond to small scaffolds inserted into larger ones. They can also result from the complex connectivity between tandemly arranged short scaffolds, or a combination thereof. Note that bubble-like structures are common but bifurcating forks are rare, meaning that there are only a few unresolved connections. This result further illustrates the strength of our dataset. A number of long scaffold chains, long-range connectivity and bubble-like structures are shown in S9–S11 Figs. Detailed examples of assembly gaps filled-in by small scaffolds are shown in S12–S14 Figs.

In summary, in this section we show that DNA-PET helps improve genome assembly by constructing large chains composed of connected scaffolds that enable the identification and correction assembly errors. From here on, our results are based on the assembly version 4.1 after re-scaffolding.

### Genome re-annotation with RNA-PET and RNA-Seq

Gene annotation is a complex process requiring high quality experimental resources and dedicated computational tools. The transcriptional start site (TSS) is an important functional feature, which is usually difficult to annotate, especially with conventional RNA-Seq, or EST mapping. Here, we used a combination of paired end RNA-Seq (PE-RNA-Seq), conventional RNA-Seq and RNA-PET datasets to re-annotate the *X*. *tropicalis* gene content.

PE-RNA-Seq based transcript assembly and mapping were carried out following a standard procedure with TOPHAT [32] and CUFFLINKS [33]. The resulting models were then combined with RNA-PET data. RNA–PET was initially a technology designed for detecting transcripts resulting from gene fusion in cancer cells [26] and recently used to derive a *de novo* annotation of *Citrus sinensis* (the sweet orange) genome [28]. RNA-PET demarcates specifically both 5’ and 3’ ends (27bp each) of all expressed full length RNA molecules, which include normal and splice variants, truncated isoforms, fusion transcripts etc. that might be derived from various conditions. The protocol used is a modified version [34] of the original method described as “GIS-PET” [26,35]. This process is summarized in S15 Fig.

A total of seven RNA-PET libraries were constructed using RNAs isolated from two larval tissues (i.e. premetamorphic stage NF54 tadpole tailfin and limb buds), and five adult tissues (i.e. brain, kidney, liver, muscle and intestine). After paired end sequencing at 2 x 36bp, the 3' end tag of the transcript, specifically recognized by a signature sequence "AACTGCTG" [34], was identified through a Smith and Waterman (SW) algorithm. Practically, each paired end sequence read (2 x 36bp) was screened first for the 3’-specific “signature” sequence, and labeled as HT (Head and Tail) if one end had found the “signature”, labeled TT (Tail Tail) if both ends had the “signature” (noise), or HH (Head Head) if neither end had the “signature” (noise). For each library, 60% to 75% of PET sequences had HT sequences, indicating that the majority of the captured PETs represent full-length transcripts (S16 Fig). Approximately 20% to 40% PET sequences are noisy HH products largely due to sequencing errors in the ‘T’ signature. We noted, however, that relaxing the SW algorithm parameters (*i*.*e*. lowering the alignment mismatch threshold) failed to rescue more HT PETs from the HH pool. Thus, this minor artifact is a direct consequence of searching a short (8bp) signature sequence, which cannot tolerate more than two bp mismatches. Subsequent analysis was carried out only using the desired HT (5’- and 3’-tag) sequence reads.

All libraries provided high quality reads (PHRED score > = 28 over > 70% of the reads). The liver library produced a limited number of PETs compared to the others, which probably reflects some specific feature of the liver transcriptome. In order to address possible sequencing bias, we also built three libraries (from intestine, kidney, liver) for sequencing on a SOLiD platform. Note that due to the SOLiD sequencing chemistry limits, the 5' and the 3' end of transcripts cannot be distinguished and thus this data can only provide transcript boundaries, irrespective of transcription orientation. However, the coverage of the transcription units with transcripts models between the two sequencing platforms was highly correlated *r* = 0.867, illustrating that the RNA-PET-based transcript models have little bias from different sequencing platforms.

PET clustering was carried out independently for each library with two sets of parameters (see Methods). The parameter set is quite stringent and is less sensitive to noise, though at the cost of producing shorter clusters. The relaxed set of parameters tends to produce longer models, but may merge together a few overlapping clusters. The two resulting datasets were merged and filtered out by selecting independently, for each transcription unit, the longest model without over-clustering. This "promotion" step adapts the stringency of the clustering parameters defined by the local context. Clusters were subsequently coalesced in order to derive new gene models.

In order to characterize the noise originating from unspecific ligation events, we plotted, for each library, the frequency of each cluster as a function of its size (in bp, with a logarithmic scale, see S17–S23 Figs). It is reasonable to assume that background noise would be associated with clusters with a low PET count, whereas PET count would be more robust for clusters derived from highly expressed genes in the tissue. In all PET libraries, the frequency of cluster size followed a bimodal distribution, with a sharp drop at 3.3 (~2Kb). When only clusters with increasing PET count were taken into account, the first peak progressively disappeared, indicating that cluster span smaller than 2Kb were associated with low PET count noise and were discarded. Putative transcript models were further filtered if they were not supported by at least 5 reads per Kb, based on strand-specific conventional RNA-Seq carried out from the same RNA samples (see Methods). On average, PETs could be clustered into 712,817 clusters (S2 Table) that may correspond to as many alternative transcripts. Putative transcript models were further coalesced and were assigned as 17,993 distinct genes.

**Fig 2:**
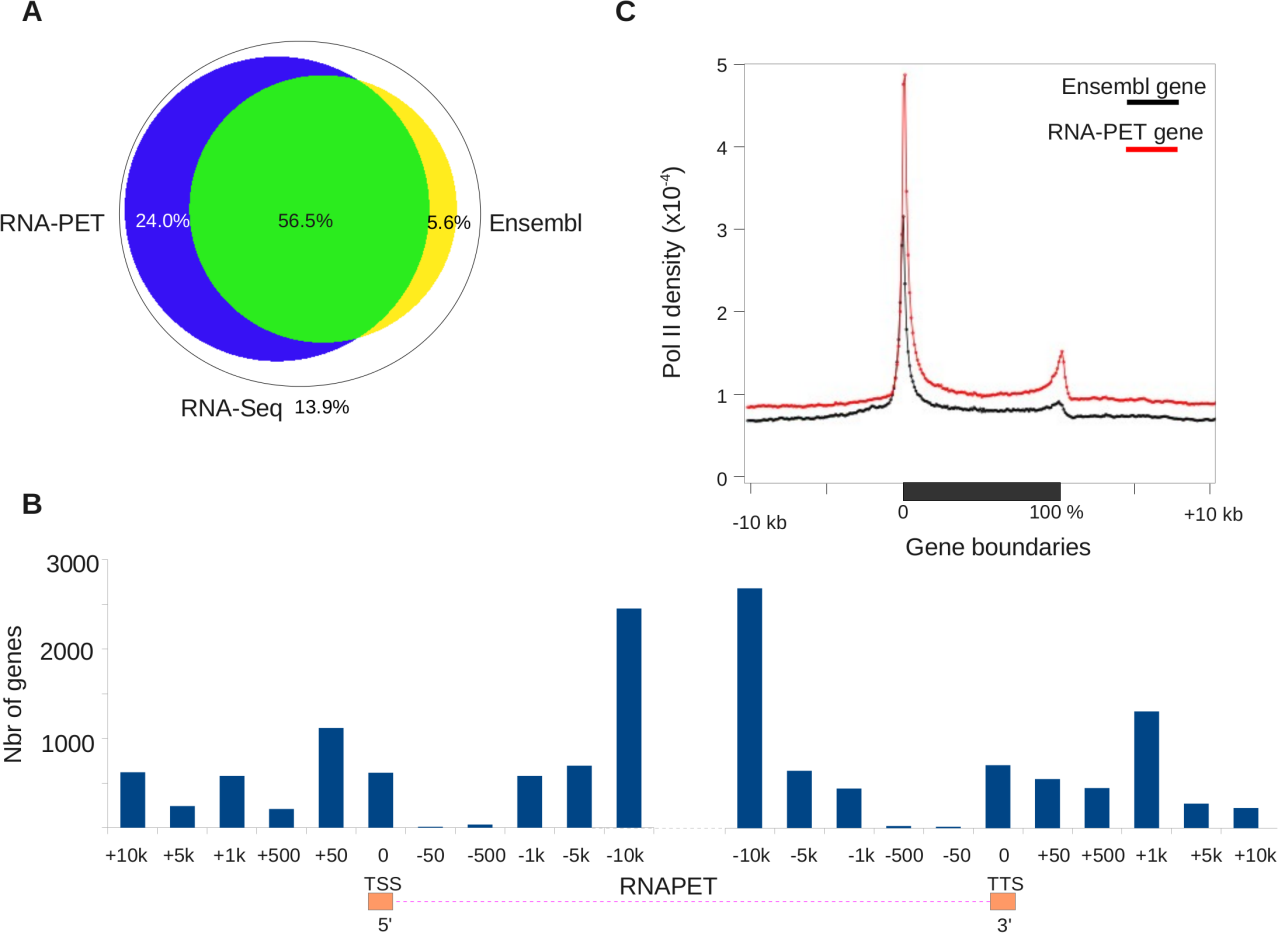
RNA-PET efficiently captures transcripts ends. A. Overlap between RNA-Seq reads and Ensembl and RNA-PET-based models. B. Demarcation of gene model boundaries by RNA-PET. The histogram shows the relative size of Ensembl gene models in bins of various sizes. C. Enrichment of RNA-Pol II around Ensembl gene models and RNA-PET-based models. This shows that RNA-Pol II density fits well with RNA-PET based models, but not Ensembl models.

Overall, the RNA-PET and RNA-Seq identified 12,387 expressed genes already annotated in the Ensembl database from the tissues tested. A total of 4,729 genes described in Ensembl were not expressed, probably because they were not expressed (or at low level) in the tissues at the developmental stages examined. Moreover, we also identified 5,607 previously unannotated transcription units and a total of 381,461 distinct transcription initiation sites. Approximately 80% of the RNA-Seq reads were located within the boundaries of the new models (Fig 2A). In contrast, around 63% of RNA-Seq reads overlap with Ensembl gene models, indicating that improvement in description/annotation is required for the existing assembly on gene and transcription units. Moreover, Ensembl models specifically capture only a marginal fraction of RNA-Seq reads. Altogether, these results showed that gene re-annotation, based on the tissue RNA sequenced, significantly improved transcript annotation. In order to further characterize the improvement of gene annotation, we plotted the density of RNA-Seq reads and the RNA-PET coverage over the normalized length of Ensembl gene models, together with 10kb upstream and downstream (see Methods and S24 Fig). RNA-Seq reads are mostly found within gene bodies, although a significant fraction of them extends beyond the gene boundaries into the 5' and 3' flanking regions. The reciprocal plot showing that the new annotation encompasses most Ensembl genes and efficiently captured RNA-Seq reads confirms this result (S24 Fig).

**Fig 3:**
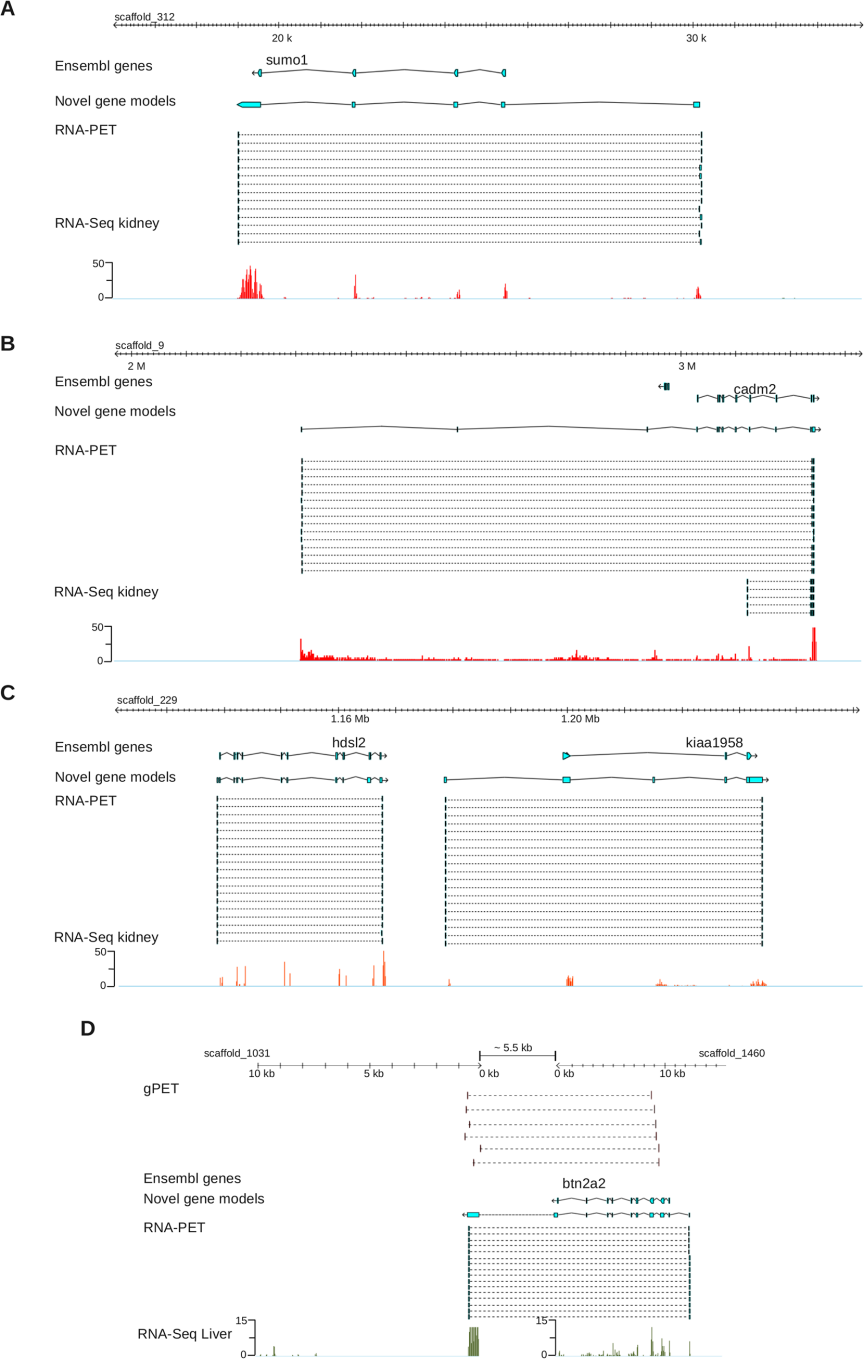
Examples of genome annotation improvements. Track order: Ensembl models, RNA-PET based models, RNA-PET ditags and RNA-Seq reads density. A, B, C: *sumo1*, *cadm2* and *kiaa1958* loci. D: Un-annotated gene split over scaffold_1031 and scaffold_1460.

Moreover, our new models are significantly longer than Ensembl gene models (average 34,306bp vs 27,688bp). We found 58% of Ensembl genes to be significantly longer (> x 1.3 or > 5Kb) once re-annotated. In order to further illustrate this point, we plotted gene length of from Ensembl versus improved annotation, again showing a strong shift toward re-annotated models (Fig 2B, S25 Fig). Importantly, we also found that RNA-PET could also capture the 5' end of 938 genes, which were missed with PE-RNA-Seq. This finding further illustrates the value of RNA-PET for precisely demarcating gene boundaries. Overall, our RNA-PET data detected a total of 187,396 TSS. A few illustrative examples of improvement are shown in Fig 3A to 3C. The *cadm2* gene is an extreme case (Fig 3B), for which the Ensembl gene model is 210Kb long, but the RNA-PET model extends the gene boundaries by an extra ~730Kb. The resulting new gene model, strongly supported by RNA-Seq data, is ~940Kb long. Gene size can vary over several orders of magnitude whereas transcript size, which is ultimately measured by RNA-PET, is much shorter, more homogeneous and thus, easier to capture. To confirm that RNA-PET efficiently captures transcripts ends, we exploited the fact that the 5' and 3' ends of transcribed genes is enriched in RNA Pol II [36] and carried out a chromatin immuno-precipitation (ChIP) with an anti-RNA Pol-II antibody. RNA Pol-II ChIp was followed by deep sequencing (ChIP-Seq), and derivation of genome-wide density profiles. As shown in Fig 2C, RNA Pol II is strongly enriched at 5' and 3' ends of RNA-PET models. Furthermore, the RNA-Pol II peak at the 5' end of genes is offset by 31bp relative to the TSS defined by RNA-PET based models (Fig 2C). This result is consistent with previous observations where genome wide profiling showed that RNA Pol II density peaks between 25 to 45bp downstream from the TSS [37]. In the case of the Ensembl gene models, the RNA Pol II profiles are also enriched at their 5' and 3' ends, but to a much lesser extent and the offset, relative to the gene model start, is much lower (< 10bp, S26 Fig). Given that RNA Pol II elongation pauses at a checkpoint located ~40bp downstream of the TSS, these results strongly support the view that RNA-PET helps capture the 5' end of genes more precisely, thereby improving gene annotation.

Interestingly, an additional ~800 RNA-PET models, well supported by RNA-Seq, were found to overlap two scaffolds, reflecting the fragmented state of the assembly. In most cases (~80%), however, the two scaffolds actually belonged to the same hyper-scaffold, as they were linked together by clusters of discordant DNA-PETs (Fig 3D). These examples cross-validated the DNA-PET and RNA-PET dataset consistency.

Altogether, these results clearly showed that (i) RNA-PET based gene models are strongly supported by RNA-Seq and RNA Pol II ChIP-Seq data, and (ii) existing gene models are extensively improved.

### Benefit of DNA-PET and RNA-PET for ChIA-PET analysis

Our overall aim at the outset of this work was to apply ChIA-PET analysis of TR binding sites to increase knowledge of the gene networks controlling metamorphosis. The details of the experimental procedure and the full datasets generated by this method applied to *X*. *tropicalis* will be published elsewhere. Here we selected salient statistics and examples to illustrate the benefit of the re-annotation process facilitated by RNA-PET, combined with the deeper analysis achieved by DNA-PET.

An initial analysis based on Ensembl models and our preliminary ChIA-PET datasets, revealed that the 5' ends of 21 genes displayed long-range interactions with distant elements. Applying our improved annotation significantly improved the resolution of this ChIA-PET analysis, increasing the number of genes with long-range interactions to 175. DNA-PET had a lower impact; with the rescue of 31 TR binding sites engaging discordant long-range interactions (*i*.*e*. connected to a functional element located on an other scaffold).

**Fig 4:**
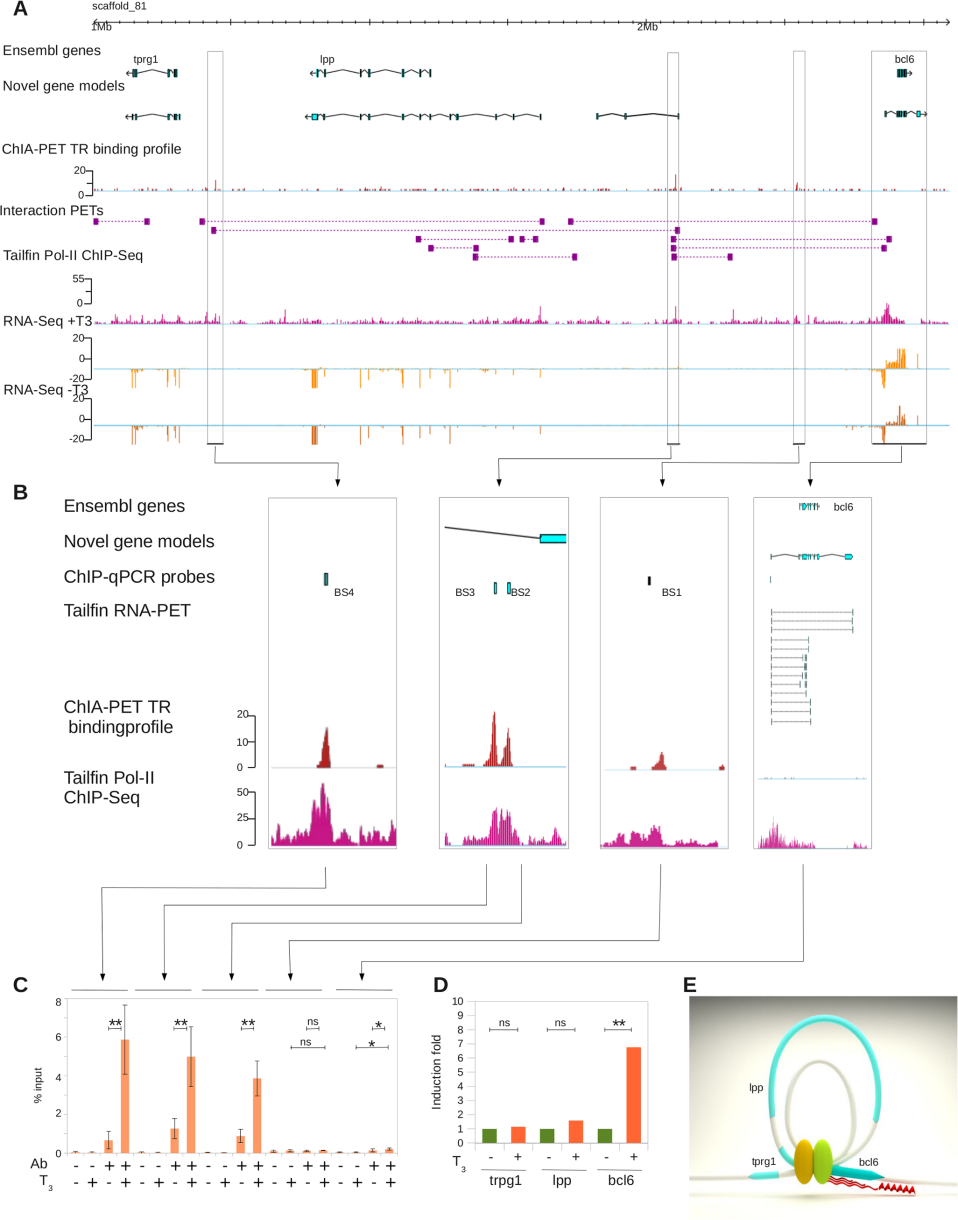
Benefit of genome re-annotation with RNA-PET for ChIA-PET analysis. A. Large genomic view of the *bcl6* locus. Track order: Ensembl genes, RNA-PET-based models, ChIA-PET TR binding density, interaction PETs, RNA Pol-II binding density, RNA-Seq reads density with (+T_3_) and without (-T_3_) treatment with thyroid hormones. B. Close up on TR binding sites. Track order: Ensembl genes, RNA-PET-based genes, location of ChIP-qPCR probes, RNA-PET ditags, TR binding density and RNA Pol-II binding density. C: ChIP-qPCR validation of TR binding at locations shown in B. Ab: Antibody, T_3_: 3’,5,3’ triiodothyronine treatment. D: Induction of *trpg1*, *lpp* and *bcl6* genes transcription assayed by RT-qPCR. E: Three-dimensional model of the locus topology.

The benefit of improving genome annotation with RNA-PET, *per se*, is best illustrated with the *bcl6* gene, the boundaries of which have been significantly extended (~10Kb) with RNA-PET (Fig 4). This gene is strongly induced by T_3_ (~ 4 fold) and there is a TR binding site (BS1) located nearby (~65Kb upstream, Fig 4). Three other TR binding sites (BS2, BS3 and BS4) are located much further upstream, 152Kb and 485Kb (BS2 and BS3 are separated by ~600bp). The binding sites BS2 and BS3 are located at the 3' end of a gene newly annotated by RNA-PET (not regulated by T_3_). Thus, based on the Ensembl annotation and ChIP-Seq-like data alone (e.g. the ChIP-Seq component of ChIA-PET), one would infer that *bcl6* transcription would be under the control of the TR bound at BS1. Interestingly, the binding sites BS2, BS3 and BS4 are connected by interaction PETs, suggesting that they physically interact with each other through DNA looping (Fig 4A). They are also connected to the 5' end of the new *bcl6* gene model, but not the Ensembl gene model. The site BS1 does not overlap with interaction PETs and does not interact with genes or other binding sites. Therefore, from this information, we infer that *bcl6* transcription would be under the control of the TR bound at BS2, BS3 and BS4, and not BS1 as initially assumed. This result is confirmed by conventional ChIP-qPCR where TR binding at BS2, BS3 and BS4 is strongly induced upon induction with T_3_, but not BS1 (Fig 4B and 4C). TR binding is also enriched at the newly annotated *bcl6* TSS to a weaker, but significant (*p* = 0.044), extent. In addition to *bcl6*, the transcripts level of the *lpp* gene is also significantly increased by T_3_ treatment (*p* = 0.044, Fig 4D). Conversely, the transcription of *trpg1* is not affected by T_3_ treatment. A model of *bcl6* locus long-range interactions through DNA looping is shown in Fig 4E.

**Fig 5:**
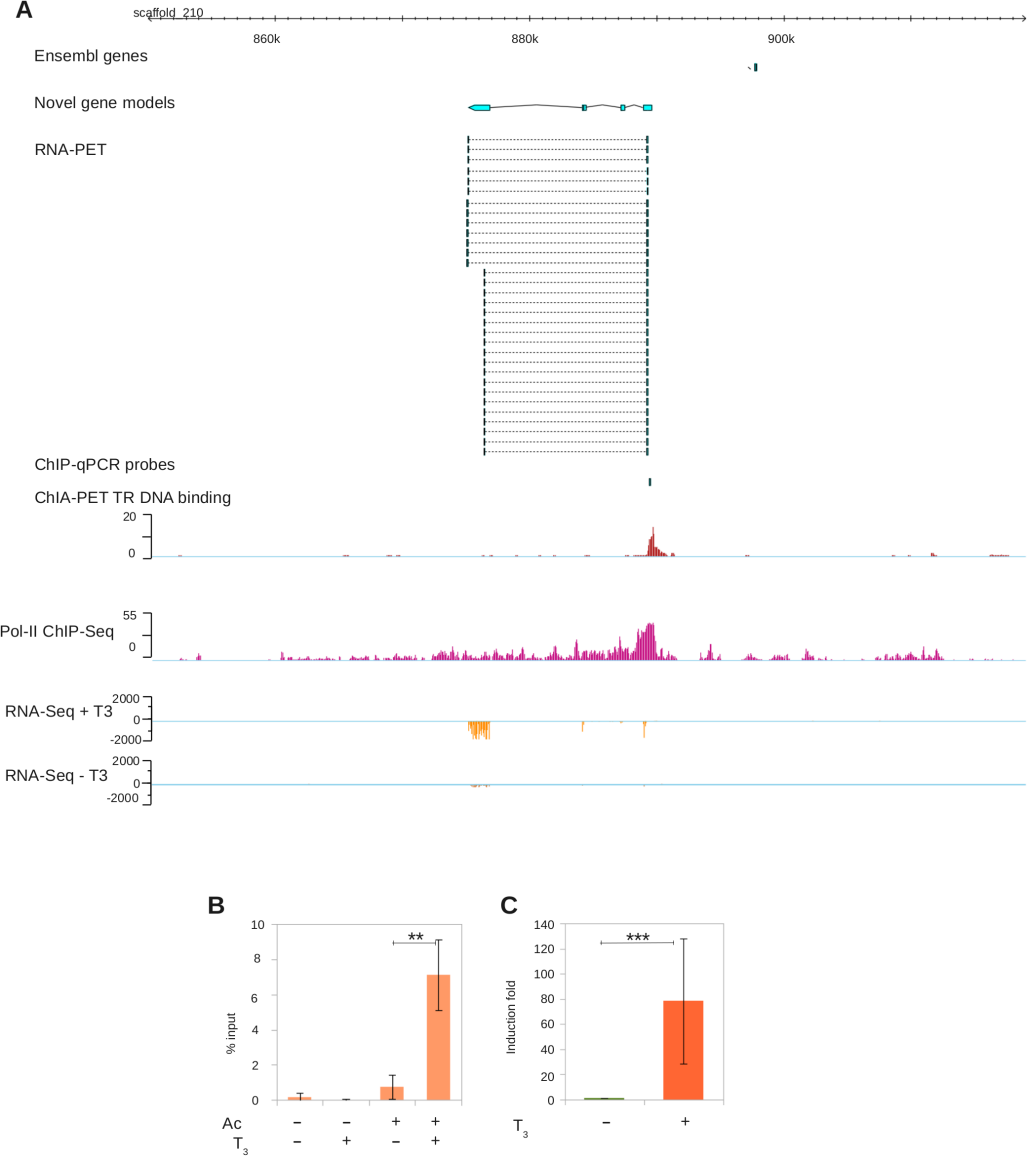
Benefit of genome re-annotation with RNA-PET for ChIA-PET analysis. A. Genomic view of an un-annotated gene. Track order: Ensembl genes, RNA-PET-based models, ChIA-PET TR binding density, RNA Pol-II binding density, RNA-Seq reads density with (+T_3_) and without (-T_3_) THs treatment. B. Close up of TR binding sites. Track order: Ensembl genes, RNA-PET-based genes, location of ChIP-qPCR probes, RNA-PET PETs, TR binding density and RNA Pol-II binding density. C: ChIP-qPCR validation of TR binding at locations shown in B. Ab: antibody, T_3_: 3’,5,3’ triiodothyronine treatment. D: Transcriptional induction assayed by RT-qPCR.

Another example of the crucial input from RNA-PET re-annotation for ChIA-PET analysis is shown in Fig 5. Before re-annotation, a single DNA-binding peak was located in a gene poor region. This DNA-bound region could not be connected to any gene because it did not engage any long-range interactions. In contrast, a novel gene, not showing up in the Ensembl track, was detected by RNA-PET (Fig 5A, top three tracks). The transcription of this novel gene is strongly induced by T_3_ treatment (~50 fold, Fig 5A, bottom tracks). ChIA-PET analysis revealed that a strong TR binding site is located precisely at the TSS. These results were further confirmed by RT-qPCR and conventional ChIP-qCR (Fig 5B and 5C).

Taken together the results demonstrate that accurate gene annotation and re-scaffolding are critical prerequisites for ChIA-PET analysis. This feature may not be an issue for genome analysis of model species, but the lack of accuracy of an assembly is a severe limitation for post-genome functional studies in non-conventional models. In this respect, combinational analysis with RNA-PET and DNA-PET proved to be a reasonably efficient way to improve gene annotation and genome assembly.

## Discussion

To better understand the molecular mechanisms controlling onset of specific transcriptional programs during metamorphosis we carried out an *in vivo* ChIA-PET analysis of TR binding sites in the *X*. *tropicalis* genome. ChIA-PET offers a key advantage over conventional ChIP-Seq analysis in that it simultaneously provides the equivalent of both ChIP-Seq plus 3C/4C analyses combined. It thus accelerates and optimizes identification of direct target genes. However, ChIA-PET and conventional functional genomic technologies require high-resolution genome annotation. Given that a sufficiently precise annotation of the *X*. *tropicalis* was not available we had to improve genome assembly and gene annotation to exploit ChIA-PET analysis. The principal finding emerging from this work is the benefit of using PET technologies for improvement of genomic resources for non-conventional models.

### PET technology: a powerful way to advance genome assembly and annotation

Sequencing technologies have dramatically improved over the past few years, increasing sequencing depth and reducing cost. The benefits of the PET technology, in improving genome assembly and annotation, are not limited to ChIA-PET analysis, but also extend to many aspects of genome analysis for which the quality of genome assembly and annotation have proved critical for genomic and post-genomic research. PET technology overcomes some of the difficulties encountered when improving existing assemblies and annotations [38]. Although many tools exist, it usually necessary to re-run complex annotation and assembly pipelines. PET technology provides a quick and easy way around this since the PET data can be used in a simple post-processing step to extend existing gene models and does not require extensive human and bioinformatic resources. A similar pipeline can be applied to the many existing (and future) draft genome assemblies for which gene annotations can be quickly improved. These technologies can also be included in the analysis pipelines used to generate *de novo* genome assemblies and gene/transcripts annotations. The description of genomic features can thus improve quickly and benefit in turn from the full power of high-resolution functional genomics, in describing the molecular mechanisms of phenotypic, genetic and epigenetic diversity.

Scientists working on conventional models may also be interested in monitoring the level of genomic divergence between the reference genome sequence and that of the cell lines/strains/individuals they are working with. They might also want to annotate the transcripts of specific tissues or cell types poorly represented in databases. Importantly, DNA-PET and RNA-PET is fairly cost-effective and do not require the combined workforce of several research institutes. This could be instrumental to bring genome projects within reach of small research communities.

### *X*. *tropicalis* genome assembly

DNA-PET allowed us to improve the published *X*. *tropicalis* genome assembly, reducing fragmentation by half, allowing estimation of gap size and correction of numerous mis-assemblies. During the course of this work, a new version of the assembly was released (v7.1), with the inclusion of a *X*. *tropicalis* genetic map. This assembly reaches chromosome-level scaffolding [39]. We note, however, that although 80% of the assembly corresponds to only 8 scaffolds, the total number of scaffolds remains large (7,730) with many small scaffolds (1,518 between 10 and 200Kb, and 6,039 < 10Kb), suggesting that many smaller-scale connections are left unresolved. Indeed, a large number of assembly gaps are exactly 100bp (17,933 out of 44,685, 40%) or 10Kb (1,200, ~3%) and may integrate smaller scaffolds in a manner very similar to the results presented above. In addition, based on our DNA-PET data, we found numerous (6,479) assembly breakpoints together with a large number of genomic inversions (1,845) of various sizes (3 to 74Kb, median 16Kb, with a vast majority at exactly 10, 20 or 40Kb). This illustrates that, despite an apparent better assembly at the chromosome level, this assembly suffers from numerous smaller scale errors. Given that this level of fragmentation (breakpoints + inversions) is greater that of version 4.1, we based our work on version 4.1. It is indeed common practice in the field of genomics to set stringent analysis criteria in order to control the noise level, often at the cost of some sensitivity.

Apparently, the improvement of genome assembly, by increasing the average scaffold size up to ~4Mb with DNA-PET, may seem less critical in the case of our ChIA-PET analysis, since the total number of rescued long-range interactions is fairly low. This is clearly not true. By using our highly curated reference we could show that the network of connectivity between regulatory regions is relatively tight and does not span regions larger than ~1Mb. This result is not trivial and supports recent data [40]. Although this may not be true for all genomes/DNA-bound proteins, physical connections among genes and regulatory elements tend to occur in genomic domains spanning tens of Kb up to a few Mb [41]. Recent work suggests that this may indeed be a general feature of transcription factors connectivity networks [18]. Thus, the fact that DNA-PET rescues a limited number of long-range interactions between functional elements may rather reflect an intrinsic property of the TR connectivity network instead of a failure of DNA-PET to rescue existing long-range interactions.

Nevertheless, in the near future, the data provided here when integrated with versions 4.1 and 7.1 of the genome will allow the Xenopus community to release a more comprehensive assembly.

### *X*. *tropicalis* genome annotation

We used an original method to improve the gene annotation of the *X*. *tropicalis* genome. RNA-PET was initially designed [26] to discover novel fusion transcripts in cancer cells and used in combination with DNA-PET analysis to detect various genome rearrangements and structural variations (such as bicistronic transcripts, transplicing transcripts, translocation generated transcripts, deletion, insertion, tandem replication-derived transcripts [42]). Originally, RNA-PET detection of fusion transcripts relied on the assumption that gene annotation is accurate and comprehensive, which is the case for human and mouse genomes. Other applications have emerged more recently, where it has been used in a reverse manner, to re-annotate a genome and re-define gene models, and allowing detection of novel genes (ENCODE, [28]). In this respect, RNA-PET has proven critical to substantially improve the definition of gene boundaries and to bring gene annotation to a level required for proper ChIA-PET analysis.

By capturing the 5' and 3' end of full-length transcripts, RNA-PET provides direct evidence for locating TSSs and TTSs. This feature was instrumental for re-defining boundaries of gene models. Indeed, transcribed genomic regions (exons) are easily identified with conventional RNA-Seq. However, without additional information, it is difficult to connect (or not) intergenic RNA-Seq signal to nearby gene. This is well illustrated in the case of a transcribed region located just upstream of a given gene model. Without additional information, it is difficult to tell whether this is a novel gene or whether this corresponds to a first exon, or that the nearby gene model should be extended. By delineating transcript boundaries, RNA-PET unambiguously solves this issue and defines cognate promoter regions. This point is crucial since most functional genomic studies focus on transcription regulation, which relies heavily on accurate and comprehensive annotation of TSSs and flanking sequences (promoter regions). Indeed, we found that the re-definition of gene boundaries with RNA-PET is a prerequisite for our analysis of ChIA-PET data. The ChIA-PET thus permits quantitative definition of physical interactions of TR with the basal transcription machinery which can show up as direct interactions between the regulator and RNA Pol II bound at the 5' end of genes, thus unambiguously identifying direct target genes. Importantly, as these physical and remote interaction sites can span over large genomic distances (> 200Kb), our results illustrate the exquisite resolution capacity of ChIA-PET in identifying transcriptional regulators even when spread over large genomic regions and at large distances from their target gene. By clearly identifying the TSS and promoter regions, this new annotation resource may be highly relevant for people working on transcriptional regulation in *X*. *tropicalis*.

It is worth noting that although RNA-PET does not provide information relative to the internal structure of transcripts, it nonetheless helps discriminate between alternative transcripts, which can share their 5' (or 3') end but not their 3' (or 5') end. Transcript reconstruction with paired-end RNA-Seq data helps document internal structure, but is not guaranteed to capture the 5' end of transcripts. Nonetheless, paired-end RNA-Seq has the advantage of being less demanding in terms of sample quantity and library construction. Thus, even when extensive RNA-Seq (conventional or paired-end followed by transcript assembly) data are available, RNA-PET is a precious complement. In addition to improve the definition of annotated genes, we could also detect numerous RNA-PET models (5' and 3' ends of transcripts) split over two scaffolds, illustrating further benefit of using RNA-PET to correct and improve the existing assembly and gene annotations.

In summary, this work provides a set of biological resources: (i) RNA-PET libraries providing better demarcation of genes, a list of alternative TSS and promoter regions, (ii) two large insert DNA-PET libraries (9Kb and 17Kb) helping re-assemble the published genome and providing the location of structural polymorphisms. The workflow also demonstrates how these resources can be exploited by the scientific community for functional, structural and evolutionary analysis of genomes in non-conventional models.

## Materials and Methods

### Ethics statement

Animal care was in accordance with institutional and national guidelines. Ethical approval for animal experimentation has been issued for this research project (ref: 68008, delivered by the Cuvier Ethic Committee).

### Animals and treatments

Adult *X*. *tropicalis* frogs (first and second generation frogs from a 5th generation inbred Nigerian strain obtained from NASCO, Fort Atkinson, WI) were maintained at 24°C in aquatic housing system (MPAquarien, Rockenhausen, Germany). Mating was induced by injection of 200U of human chorionic gonadotropine for females and 100U for males (Chorulon; Intervet, Beaucouze, France). Tadpoles were raised at 26°C. Tadpoles were staged according to the normal table of *Xenopus laevis* (Daudin) of Nieuwkoop and Faber (NF) (1967) [43]. For THs treatment, tadpoles at stage NF54 were exposed 24h, in 5 liters with 10nM T_3_ (ref: T2752; Sigma, St. Quentin Fallavier, France). Tadpoles were killed after anesthesia (ref: E10505; 0.01% MS222, Sigma) before hindlimb and tail fin dissection.

### RNA isolation

For each physiological condition, tissues were isolated from groups of 10 tadpoles, collected, flash frozen, and stored at -80°C. Tissue lysis was performed in 500 μl of RNAble (ref: Gex-ex-T00-0U; Eurobio, Les Ulis, France) with one bead (INOX AISI 304 grade 100 AFBMA) using Tissue Lyser II apparatus (Qiagen, Courtaboeuf, France) for 1min at 30 Hz. The lysed tissues were mixed with chloroform and incubated on ice for 5 min before centrifugation (12,000 x g, 15min, 4°C). The supernatant was subjected to RNA purification with RNeasy MinElute Cleanup kit according to manufacturer (ref: 74,204; Qiagen). For Adult tissues, 2 to 50 frogs were required (based on the mass of tissue removed). Tissues were isolated, flash frozen, and stored at -80°C. Tissue lysis was performed in 5 ml of RNAble with an ultra turrax. The lysed tissues were mixed with chloroform and incubated on ice for 5 min before centrifugation (12,000 x g, 15min, 4°C). The supernatant was subjected to RNA purification with RNeasy MidiElute Cleanup kit according to manufacturer (ref: 75,142; Qiagen). RNA concentration was measured by optic density. RNA quality was estimated by microcapillary electrophoresis using Qiaxcel (Qiagen).

### RT-qPCR analysis

Potential contamination by genomic DNA was removed using DNAse treatment as described by the provider (ref: AM1907; Turbo DNA free; Ambion, LifeTechnologies, Courtaboeuf, France). Reverse transcription (RT) was done as previously described [44] using Superscript III reverse transcriptase (ref: 18,080,044; LifeTechnologies, Fisher Scientific, Illkirch, France). RT products were analyzed by real-time qPCR performed on an ABI 7300 (Applied Biosystems). Primers were designed using Primer express (Applied Biosystems). The list of used primers is given in S3 Table. Prism 7300 system software (Applied Biosystems) was used to analyze the results. Data are presented in means of Log(2ΔΔCT) and SEM (CT, cycle time). The raw data of three biological replicates are first normalized on the endogenous control gene rpl8 (ΔCT: mean CT rpl8 minus mean CT gene of interest) followed by Log transformation, so the variances between groups succeed the t-test. For statistical analysis, the Log of the normalized data was subjected to a one-sample two-tailed t test (α = 5%).

### Processing of RNA-Seq data

Conventional RNA-Seq was carried out on SOLiD plateform (Lifetechnologies) following the manufacturer instructions and reads were mapped using Bioscope pipeline. Paired-end RNA-Seq was carried out by SERVICEXS (www.servicexs.com, Leiden, Netherlands) on the Illumina HiSeq plateform. Reads were processed with Tophat (v2.0.6) and Cufflinks (v2.0.2) run with default parameters, except for the Tophat r parameter set to 100.

### Construction of RNA-PET libraries

A 27bp DNA tag sequence from each end of the full-length cDNA was then extracted after a type III restriction enzyme (*Eco*P15I) digestion. The resulting Paired End diTag fragment (27bp-linker-27bp) was ligated to sequencing adaptors at both ends, amplified by PCR, and purified as templates for paired end (PE) sequencing with Illumina system. Construction of the RNA-PET genomic libraries (S15 Fig) was using an improved and modified protocol version to the previous “GIS-PET”, which had been published and described in [35]. Briefly, full length polyA mRNA were purified from high quality total RNAs with MACs polyT columns. Approximately one to two micrograms of purified mRNA were used for reverse transcription into full-length cDNA, which were then biotinylated at the ends. Double stranded cDNAs were then circularized after ligation to specific DNA linker sequences. The 5’ and 3’ ends (27–27bp) tags were then extracted through EcoP15I digestion and resulting PET templates [5’-27bp-linker-27bp-3’] which were purified from mixture by binding to streptavidin magnetic beads. Illumina sequencing adaptors were then ligated at both ends and PCR-amplified and sequenced by paired end reads at 2 x 36bp long. Reads quality was assessed with standard quality controls. The modified version of the RNA-PET construction has two major changes: (i) it generates longer (27/27bp, in contrast to the previous 18/20bp) paired end tags from 5’ and 3’ ends of transcripts, by using a type III restriction enzyme EcoP15I, (ii) eliminated bacterial-cloning procedures.

### Processing of RNA-PET and PE-RNA-Seq data

For each library, paired reads were independently mapped using bowtie version 0.12.7 [45], with stringent parameters (l = 22, n = 0, m = 1). After merging, mate pairs were subject to a stringent filter, so that only those for which both end map at a unique genomic location are retained. The orientation of transcript was resolved by looking for the three prime end-specific signature AACTGCTG in both mate pair with a Smith and Waterman algorithm [46]. Only pairs with a 3' end signature at one end were kept for further processing. Clustering was carried out by using a simple greedy algorithm aggregating together PETs sharing overlapping 5' end tags. Models unsupported by RNA-Seq reads (<5 reads per Kb) were discarded. The final models were further size filtered to reduce background noise originating from small artifactual models (see main text).

All these were carried out with simple python scripts, cython and a C library (available on request). For the sake of parallelization and reduction of computing time, we relied heavily on a 'divide and conquer' approach, which is based on a specific property of the assembly version 4.1. Indeed, the scaffolds size decreases slowly from largest to smallest (scaffold_1 to scaffold_20095). For each mapped read, we used the last two digits of the scaffold name ('_1' treated as 01 and so on) as a key to store the entry in the corresponding file. This way, data from scaffold_1, scaffold_101, scaffold_301 … are stored in file "01", data from scaffold_2, scaffold_202, scaffold_302 in file "02" … This has the benefit of splitting the datasets in 100 files of ~ equal size and thus leads to approximately equivalent running time for each processing step.

### Gene re-annotation

The annotation of the gene set was improved by integrating paired end RNA-Seq to Ensembl models, with the MAKER2 and EVM pipelines, following guidelines available from the respective web sites (http://gmod.org/wiki/MAKER, http://evidencemodeler.sourceforge.net/).

### Antibodies

The TR antibody has been previously described for use in ChIP assay [44]. RNA PolII antibody (CTD4H8) was from Epigentec (A-2032; Euromedex, Souffelweyersheim, France).

### ChIP and qPCR analysis

Up to 30 to 50mg dissected tissues from seven euthanized tadpoles were used to isolate chromatin. ChIP was done as previously described with slight modifications [44]. ChIP products were analyzed by real-time qPCR as previously described [44]. The results were expressed as percent of input and presented as means SEM of at least three independent experiments. Statistical analyses were performed with a paired two-tailed t test (α = 5%). The list of used primers is given in S3 Table. For ChIA-PET analysis, chromatin was isolated from a batch of 8 samples and subject to ChIP and ChIA-PET procedure [20]. For the RNAPol II ChIP-Seq, ChIP was carried out on chromatin extract prepared from a batch of 20 samples. The ChIP product was purified by phenol chloroform extraction with Phase lock gel and ethanol precipitation. Sequencing of the RNA Pol II ChIP product was outsourced SERVICEXS (Leiden, Netherlands).

### Preparation of genomic DNA

Genomic DNA was prepared from liver and kidney of seven male and female adults. Tissues were dissected and directly disorganized with Qiagen Tissue Lyser (30 Hz, 20 sec.) and the lysate was cleared with Qiagen QIAshredder columns (ref: 79,654). Total DNA was extracted with DNA AllPrep DNA/RNA Mini columns (ref: 80,204). All procedures were carried out according to the manufacturer's instructions.

### Generation of DNA-PET libraries

To construct large insert DNA-PET libraries (S1 Fig), whole genome DNA was randomly hydrosheared, blunt end repaired, biotinylated and ligated to a specific DNA linker sequence at both ends. The linker-modified genomic DNA fragments were then gel-purified to obtain the size range (e.g., 10Kb, or 15Kb) of interest, followed by circularization with linker ligation. The paired end tags (PET at 27/27bp) were then excised through a type III restriction enzyme EcoP15I digestion. Extracted PETs were purified by binding to streptavidin magnetic beads, followed by ligation with SOLiD-specific adaptors at both ends. The complete sequencing template structure of the DNA-PET is amplified by PCR and processed for SOLiD mate-pair sequencing. Reads quality was assessed with standard quality control procedures.

### DNA-PET data processing

The re-scaffolding workflow is composed of four main steps: reads mapping, identification of mis-assemblies and scaffold splitting, the identification of repeated regions and re-scaffolding *per se*, through a graph-based approach.

#### Data cleaning and mapping

The two DNA-PET (mate-pair) libraries were mapped using the SOLiD Corona-Lite pipeline, allowing 2 mismatches for each 25bp tag. The Corona-Lite mate-rescue step was carried out allowing up to maximum 4 mismatches in a read. The details of the pairing statistics are summarized in S1 Table. The pipeline also classified PETs as concordant PETs (cPETs) or discordant PETs (dPETs). cPETs were defined as those PETs where both tags mapped to same reference, same strand, in the correct 5' to 3' ordering and within expected span range. The PETs which were rejected by cPET criteria were classified as dPETs, which thus combine a mixture of PETs: those mapping on different references and those mapping on the same reference, but with discrepancy relative to the expected span range.

#### Scaffold splitting

The insert size of concordant PETs for the 10Kb library (IXT010) range from 5,997–11,961bp. For the 17Kb library (IXT011), the insert size is between 9,318 and 22,026bp. We analyzed the concordant PET coverage across the reference genome to determine possible mis-assemblies ('assembly breakpoints'). They were defined as regions where the concordant PET fragment coverage drops to 0 (*i*.*e*. their left and right hand sides are un-connected). Scaffolds were split into two at assembly breakpoints. This resulted in 19,847 scaffolds. Furthermore, we split the resulting scaffolds at assembly gaps (blocks of 10,000 or more ‘N’s, provided there are no PET spanning over these regions) in order to replace them by other scaffolds. Splitting scaffolds at smaller assembly gaps (between 5,000 and 9,000 blocks of 'N's) leads to a much-fragmented assembly, which fails to re-scaffold properly. This is not unexpected because the fragments are shorter than the insert size of the libraries. This scaffold splitting step resulted in a total of 21,909 scaffolds. These steps are important since they significantly improve the connectivity between scaffolds and provide a much-increased map of scaffolds ordering (see Results).

#### Identification of repeated regions

Since the presence of repeated sequences can interfere with the re-scaffolding process, regions, repeated regions were identified based on their high PET coverage (>500 tags per 1Kb window) and discarded. This cut-off is based on the global PET tag density distribution and is susceptible to vary depending on sequencing depth, the repeat content, the nature of repeated sequences and as well as other model-dependent parameters.

#### Graph-base re-scaffolding

This step consists of connecting distinct scaffolds connected by a set of discordant PETs. Importantly, the resolution of this step is limited by the size of the DNA fragments used to build the libraries. Therefore, scaffolds separated by more than ~22Kb (the maximum span of the 17Kb library) won't be connected together. Also, our 10Kb and 17Kb libraries are not suitable for re-scaffolding the numerous small un-assembled sequencing reads (< 5Kb) of the version 4.1 assembly. In the graph, nodes represent scaffolds and edges represent dPETs, weighted by the number of independent (but non redundant) PETs. Edges with a weight ≤ 3 were discarded in order to filter-out low confidence background. Each library (IXT010 and IXT011) was processed separately, leading to two distinct sets of edges. For keeping track of the splitting process at assembly breakpoints, we introduced extra edges with weight 1 connecting the two scaffolds that were split at a blocks of ‘NNN’ (see above).

The end result of the re-scaffolding process is a set of ordered scaffolds, which correspond to a set of path through the graph. It is computed by attributing a score to each alternative path, by summing the weight of all of edges supporting it, minus the total weight of conflicting edges. A heuristic is used to find the path that maximizes the score for each scaffold.

Although we did not use it, it is possible to estimate the size of the un-sequenced region between re-connected scaffolds. For the details of the algorithm, please refer to [30].

### ChIA-PET analysis and processing of ChIA-PET data

The ChIA-PET principle, technical procedures and analysis are already described in [16] and [20]. Cross-linked chromatin complex (DNA/Protein) are fragmented by sonication to a given size range (~200–600bp), and enriched by chromatin immunoprecipitation (ChIP) method with a specific antibody of interest. In the enriched chromatin complexes, tethered DNA fragments (spatial located) are joined together by specifically designed DNA linkers through “proximity ligation”, and joined DNA fragment are then extracted for PET template, followed by high-throughput NGS sequencing (Illumina). PET sequences are mapped to a reference genome, which can reveal genome-wide protein/DNA relationship of (i) binding sites (like CHIP-Seq data), and (ii) long-range chromatin interaction relationships between two remote loci brought together by protein(s) at the interest.

## Supporting Information

S1 Fig Principle of DNA-PET. Genomic DNA is fragmented in 10 or 17Kb fragments and circularized with adaptors containing a EcoP15I restriction site (pale blue). After restriction with EcoP15I, released DNA fragments are ligated to sequencing adaptors (green), sequenced and mapped on the reference genome. cPETs (deep blue) correspond to PETs where the two tags map on the same scaffold whereas dPETs (red) correspond to PETs where the two ends map on different scaffolds. dPETs can be used to improve scaffolding. The 5' and 3' ends of sheared genomic DNA fragments are colored in purple and orange, respectively.
(PDF)

S2 Fig Quality control of the DNA-PET datasets. A. Insert size distribution of DNA-PET libraries of 9.6Kb (IXT010) and 17Kb (IXT011), estimated as the average genomic span of cPETs (blue line). B. Correlation of coverage density between DNA-PET libraries IXT010 and IXT011, computed by scoring the number of cPETs overlapping successive 20Kb windows. The tight distribution indicates that the genome coverage derived with two independent libraries is highly correlated.
(PDF)

S3 Fig Discrepancy between expected and annotated gap size, for '50bp gaps'. The difference between the expected and the measured span of individual cPETs has been used to estimate the length of '50bp gaps'. This measure of discrepancy is positive for cPETs overlapping a region that is actually larger than 50bp and conversely for negative values. The red line represents the average value.
(PDF)

S4 Fig Examples of discrepancy between expected and measured assembly gap length, for 50bp gaps. Individual cPETs spanning a single assembly gap of 50bp have been used to estimate their actual length. For each cPET, the measured span size (left panel) was compared to the expected span size. Disagreements between expected and measured span sizes were used to derive independent estimate of the gap actual size (right panel). The number of cPETs, the average and median of the estimates are shown. A, B, C are three illustrative examples.
(PDF)

S5 Fig Example of structural polymorphism at assembly gaps of 50bp. In a number of cases, the measure of the actual gaps length follows a bimodal distribution, with two populations labeled α and β. See legend of S4 Fig for details. A, B and C are illustrative examples.
(PDF)

S6 Fig Example of discrepancy between expected and measured assembly gap length, for assembly gaps other than 50bp-long. Individual cPETs spanning a single assembly gap (≠ 50bp) have been used to estimate their actual size. See legend of S4 Fig for details. The number of cPETs, the expected size, the average and median of the estimates are shown. A, B, C are three illustrative examples.
(PDF)

S7 Fig Example of structural polymorphism at assembly gaps ≠ 50bp. In a number of cases, the measure of the actual gaps length follows a bimodal distribution, indicated α and β. See legend of S4 Fig for details. A, B and C are illustrative examples.
(PDF)

S8 Fig Example of assembly breakpoints. Genomic views two assembly break points. Track order: Assembly gaps, clusters of dPETs for DNA-PET libraries IXT010 and IXT011, cPETs coverage profile computed from libraries IXT010 and IXT011. The average insert size for these libraries is 9.6Kb and 17Kb, respectively. Numbers above assembly gaps indicate their length. Values in brackets correspond to their estimated length based on cPETs datas, when available. Assembly gaps of 50bp are shown in red. Assembly breakpoints potentially occur when cPET coverage drops to zero, meaning that the two sides are not connected together, at the resolution of the DNA-PET libraries.
(PDF)

S9 Fig Example of long range DNA-PET connectivity and re-scaffolding. Scaffolds are shown as ovals, indicating their number (*e*.*g*. 291 for scaffold_291). The dashed line corresponds to the path through the graph and represents the final ordering of the scaffolds relative to each other. Colored lines correspond to the connection and numbers indicate the estimated distance between scaffolds and well as dPETs support. Colors are heat map coded. In this example, a few small scaffolds (scaffold_7625, scaffold_23017, scaffold_7932) are nested between longer ones.
(PDF)

S10 Fig Few examples of re-scaffolding. Scaffolds are shown as ovals, indicating their name. Colored lines correspond to the connection and numbers indicate the dPETs support. Colors are heat map coded. Bubble-like structure correspond to small scaffolds inserted into larger ones or result from the complex connectivity between tandemly arranged short scaffolds.
(PDF)

S11 Fig Example of small scaffolds inserted into assembly gaps of larger ones. Scaffolds' fragments are shown as ovals, indicating their name (*e*.*g*. 291 for scaffold_291), which part of the original scaffold they correspond to, and their size. The dashed line corresponds to the path through the graph and represents the final ordering of the scaffolds relative to each other. Colored lines correspond to the connection and numbers indicate the dPETs support. Colors are heat map coded. A and B represent two examples of small scaffolds being inserted into assembly gaps present in larger ones.
(PDF)

S12 Fig Example of assembly gap fill-in by a smaller scaffold. A. Conceptual representation of the connectivity between scaffolds_1883 and scaffold_2. Numbers above the links indicate the cPET count for each library. Clusters of cPETs connecting the left and the right side are drawn in yellow and orange, respectively. B, C. Detailed view of the entire scaffold_1883 and a sub-region of scaffold_2. The last two tracks correspond to the coverage density of cPET, for each DNA-PET library.
(PDF)

S13 Fig Example of assembly gap fill-in by a smaller scaffold. A. Conceptual representation of the connectivity between scaffolds_12632 and scaffold_11. Numbers above the links indicate the cPET count for each DNA-PET library. Clusters of cPETs connecting the left and the right side are drawn in yellow and orange, respectively. B, C. Detailed view of the entire scaffold_12632 and a subregion of scaffold_11. The last two tracks correspond to the coverage density of cPET, for each DNA-PET library. Note that the ~8Kb assembly gap on scaffold_11 is small compared to the average insert size of the two libraries. As a result, cPET coverage does not drop to zero.
(PDF)

S14 Fig Example of complex gap fill-in by smaller scaffolds. A. Conceptual representation of the connectivity between scaffolds. Numbers above the links indicate the cPET count for each DNA-PET library. Clusters of cPETs connecting the left and the right side of the nested scaffolds are drawn in yellow/blue and orange/green. B, C, D. Detailed view of the nested scaffolds and a sub-region of scaffold_1. The last two tracks correspond to the coverage density of cPET, for each DNA-PET library. Note that the ~8Kb assembly gap on scaffold_1 is small compared to the average insert size of the two libraries. As a result, cPET coverage does not drop to zero.
(PDF)

S15 Fig Principle of RNA-PET. Full-length polyA+ RNA are used as template to generate full-length cDNAs. Double stranded adaptors specific for the 5' and 3' ends (pink, orange) are ligated to the ends of the transcripts. Adaptors contain an EcoP15I restriction site, cutting 27bp away from its binding site. After circularization and restriction with EcoP15I, sequencing adaptors are ligated to the resulting Paired-End diTag, prior to sequencing, mapping and clustering.
(PDF)

S16 Fig Statistics of the identification of the 3'-end signature in RNA-PET di-tags. The adaptor at the 3' end of transcripts is labeled with a specific signature (AACTGCTG). RNA-PET di-tags are labeled HT (Head and Tail), TT (Tail Tail) or HH (Head Head) on whether the signature was found at one end, both or none, respectively. Venn diagrams represent the proportion of HT, HH and TT di-tags obtained for each tissue library. Numbers indicate the total number of PETs per category.
(PDF)

S17 Fig Distribution of the genomic span of individual RNA-PET clusters in adult brain. Blue, yellow and red lines denote ~1.5, ~2.0 and ~2.5Kb, respectively.
(PDF)

S18 Fig Distribution of the genomic span of individual RNA-PET clusters in adult liver. Blue, yellow and red lines denote ~1.5, ~2.0 and ~2.5Kb, respectively.
(PDF)

S19 Fig Distribution of the genomic span of individual RNA-PET clusters in adult intestine. Blue, yellow and red lines denote ~1.5, ~2.0 and ~2.5Kb, respectively.
(PDF)

S20 Fig Distribution of the genomic span of individual RNA-PET clusters in adult skeletal muscles. Blue, yellow and red lines denote ~1.5, ~2.0 and ~2.5Kb, respectively.
(PDF)

S21 Fig Distribution of the genomic span of individual RNA-PET clusters in adult kidney. Blue, yellow and red lines denote ~1.5, ~2.0 and ~2.5Kb, respectively.
(PDF)

S22 Fig Distribution of the genomic span of individual RNA-PET clusters in tadpole tail fin skin. Blue, yellow and red lines denote ~1.5, ~2.0 and ~2.5Kb, respectively.
(PDF)

S23 Fig Distribution of the genomic span of individual RNA-PET clusters in tadpole limbs. Blue, yellow and red lines denote ~1.5, ~2.0 and ~2.5Kb, respectively.
(PDF)

S24 Fig RNA-PET significantly extends gene models. A. Density distribution of RNA-Seq reads and RNA-PET ditags over Ensembl gene models. B. Density distribution of RNA-Seq reads and Ensembl genes boundaries over RNA-PET based gene models. Gene coverage is expressed in percent of gene length.
(PDF)

S25 Fig RNA-PET significantly extends gene models. The plot corresponds to the size of individual RNA-PET-based gene models relative to their Ensembl counterpart. Gene models of the same size between the two datasets would follow the red line.
(PDF)

S26 Fig Enrichment of RNA-Pol II at the 5' end of gene models improved by RNA-PET. The RNA-Pol II density was computed at the 5' end of gene models, based on Ensembl and RNA-PET. The 5' end of Ensembl models is located 60bp downstream of the RNA-Pol II peak (red curve), typically located ~25–45pb downstream the TSS. In contrast, the 5' end of models enriched with RNA-PET data extend 32bp upstream of RNA-Pol II peak.
(PDF)

S1 Table DNA-PET pairing statistics. (XLS)

S2 Table RNA-PET clustering statistics. (XLS)

S3 Table Primers list. (XLS)
