# Supplementary figures and images for: Xenopus tropicalis Genome Re-Scaffolding and Re-Annotation Reach the Resolution Required for In Vivo ChIA-PET Analysis
Source: PLoS One. 2015 Sep 8;10(9):e0137526. doi: 10.1371/journal.pone.0137526 (PMC4562602; doi:10.1371/journal.pone.0137526)

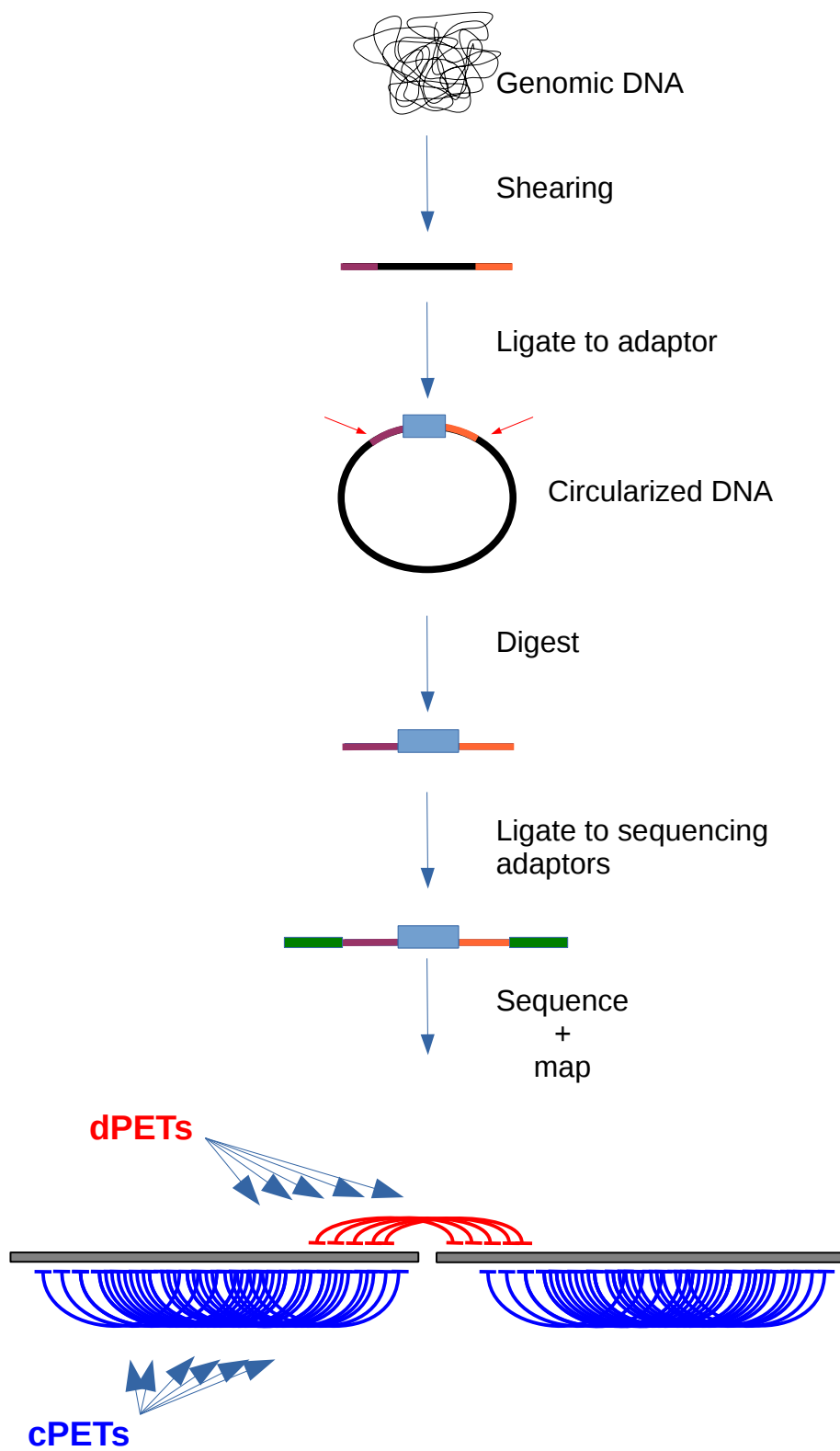

Supplement: S1 Fig — Genomic DNA is fragmented in 10 or 17Kb fragments and circularized with adaptors containing a EcoP15I restriction site (pale blue). After restriction with EcoP15I, released DNA fragments are ligated to sequencing adaptors (green), sequenced and mapped on the reference genome. cPETs (deep blue) correspond to PETs where the two tags map on the same scaffold whereas dPETs (red) correspond to PETs where the two ends map on different scaffolds. dPETs can be used to improve scaffolding. The 5' and 3' ends of sheared genomic DNA fragments are colored in purple and orange, respectively. (PDF) [file pone.0137526.s001.pdf]

**A**

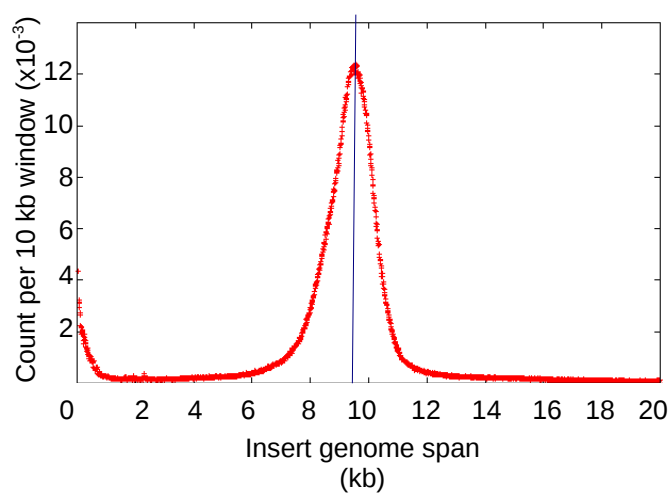

Average insert size IXT010 : 9.6 kb

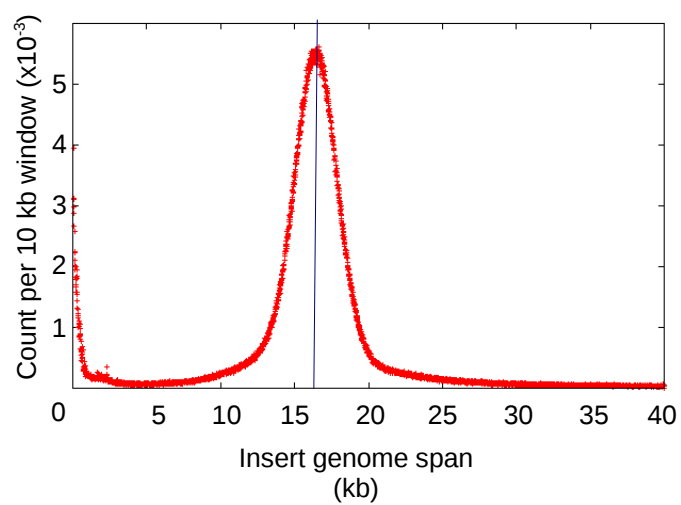

Average insert size IXT011 : 17.5 kb

**B**

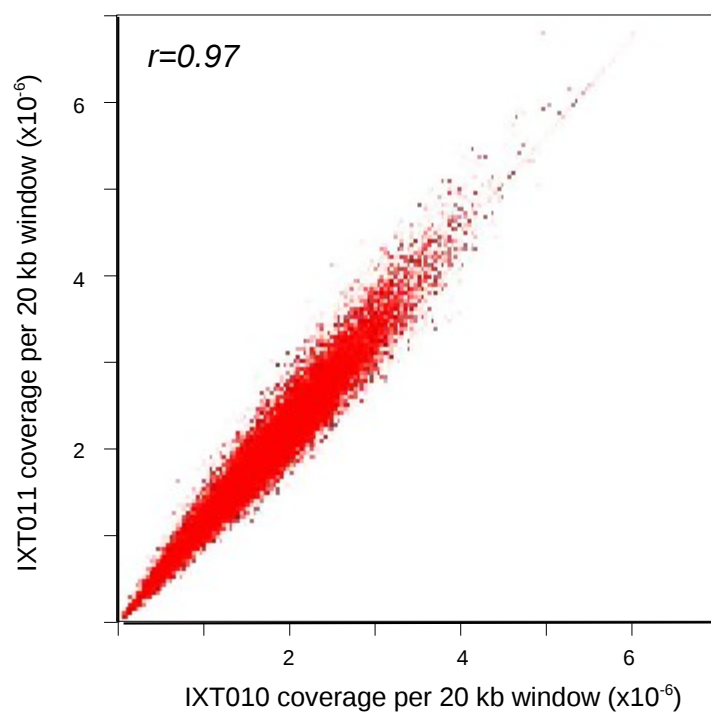

Supplement: S2 Fig — A. Insert size distribution of DNA-PET libraries of 9.6Kb (IXT010) and 17Kb (IXT011), estimated as the average genomic span of cPETs (blue line). B. Correlation of coverage density between DNA-PET libraries IXT010 and IXT011, computed by scoring the number of cPETs overlapping successive 20Kb windows. The tight distribution indicates that the genome coverage derived with two independent libraries is highly correlated. (PDF) [file pone.0137526.s002.pdf]

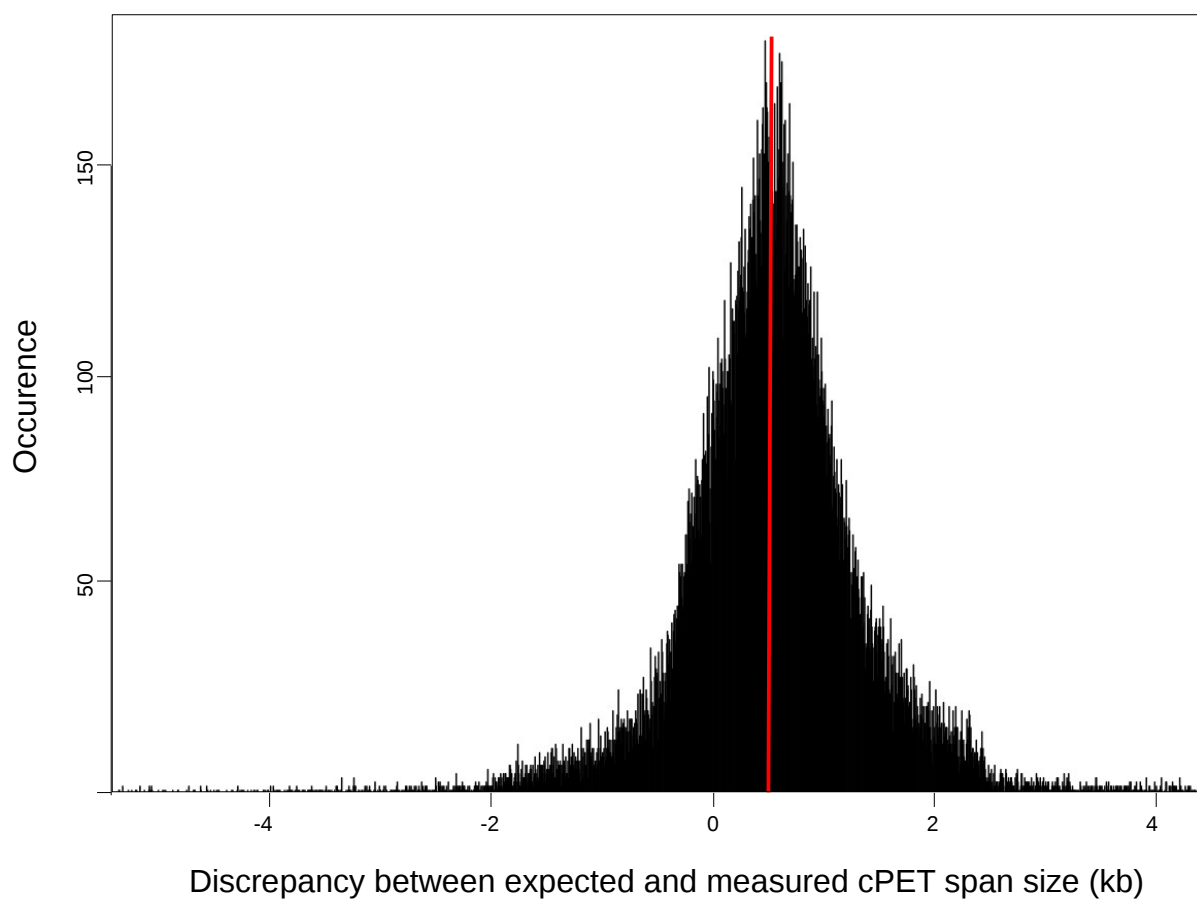

Supplement: S3 Fig — The difference between the expected and the measured span of individual cPETs has been used to estimate the length of '50bp gaps'. This measure of discrepancy is positive for cPETs overlapping a region that is actually larger than 50bp and conversely for negative values. The red line represents the average value. (PDF) [file pone.0137526.s003.pdf]

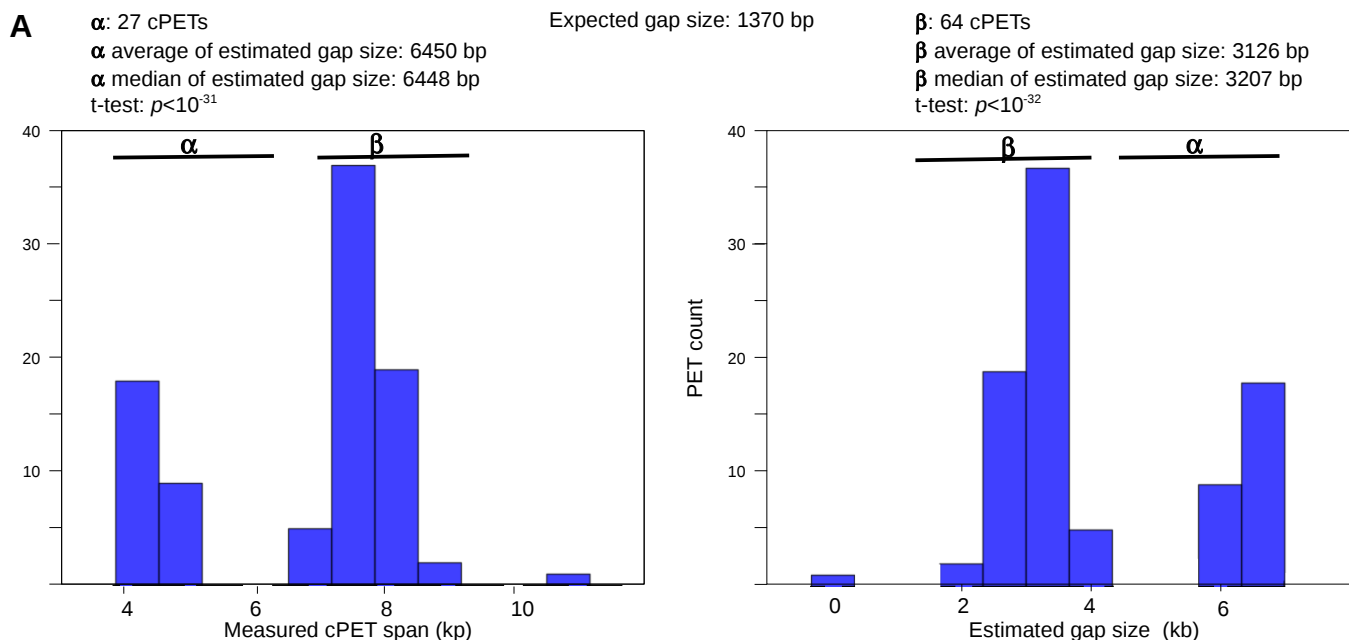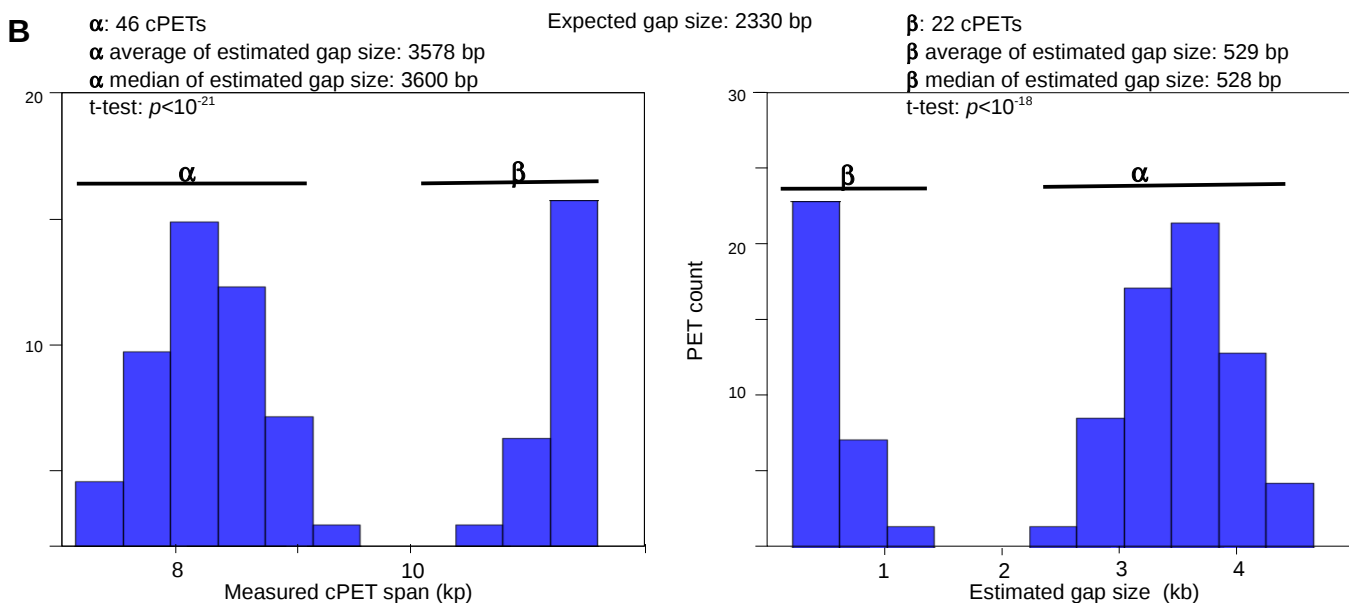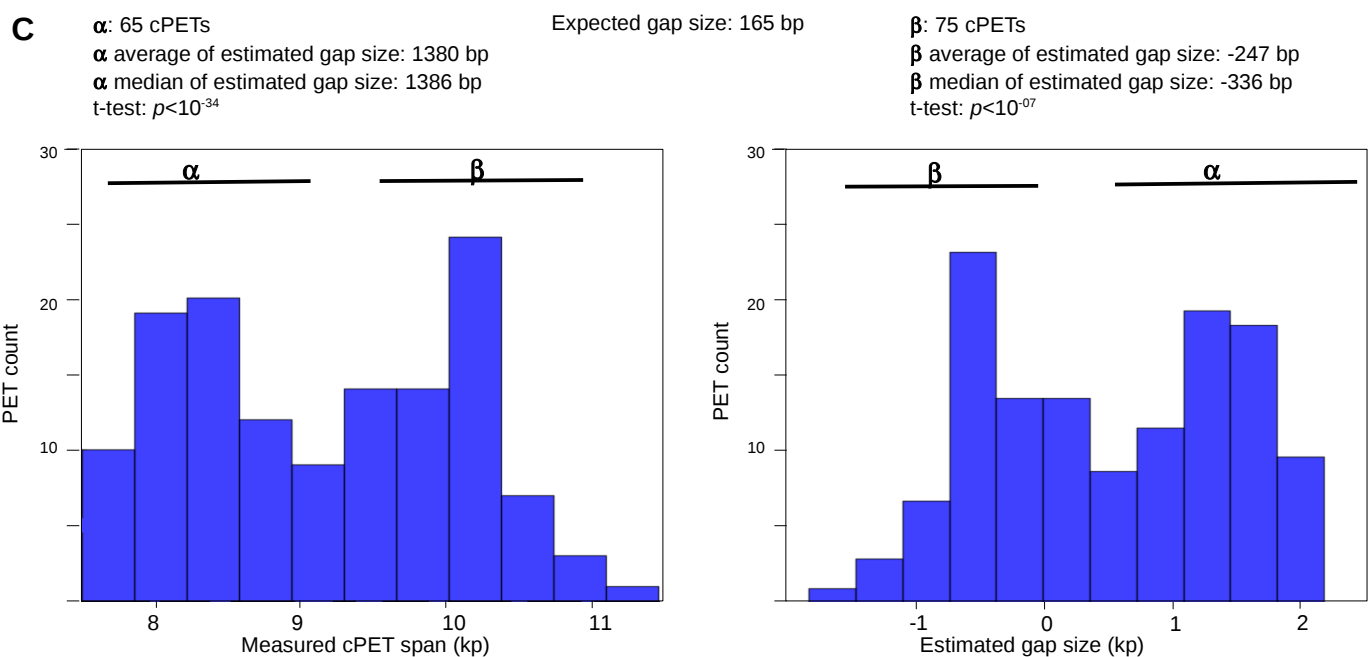

Supplement: S7 Fig — In a number of cases, the measure of the actual gaps length follows a bimodal distribution, indicated α and β. See legend of S4 Fig for details. A, B and C are illustrative examples. (PDF) [file pone.0137526.s007.pdf]

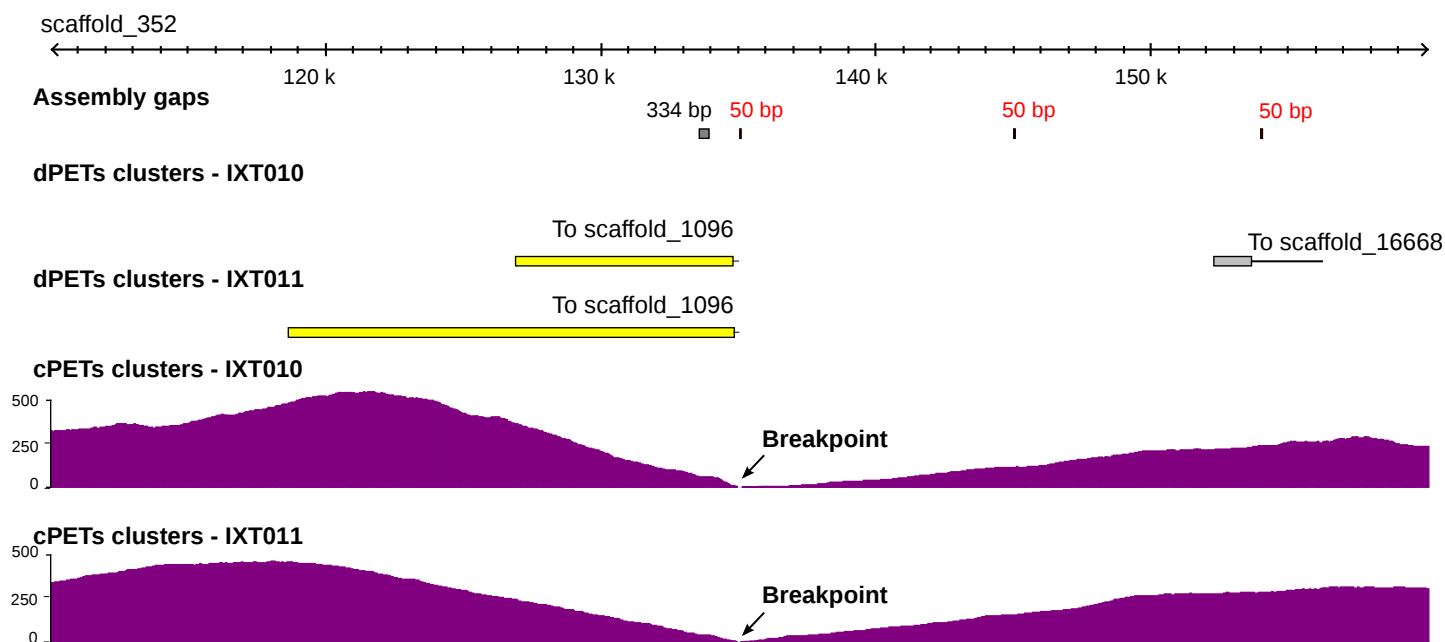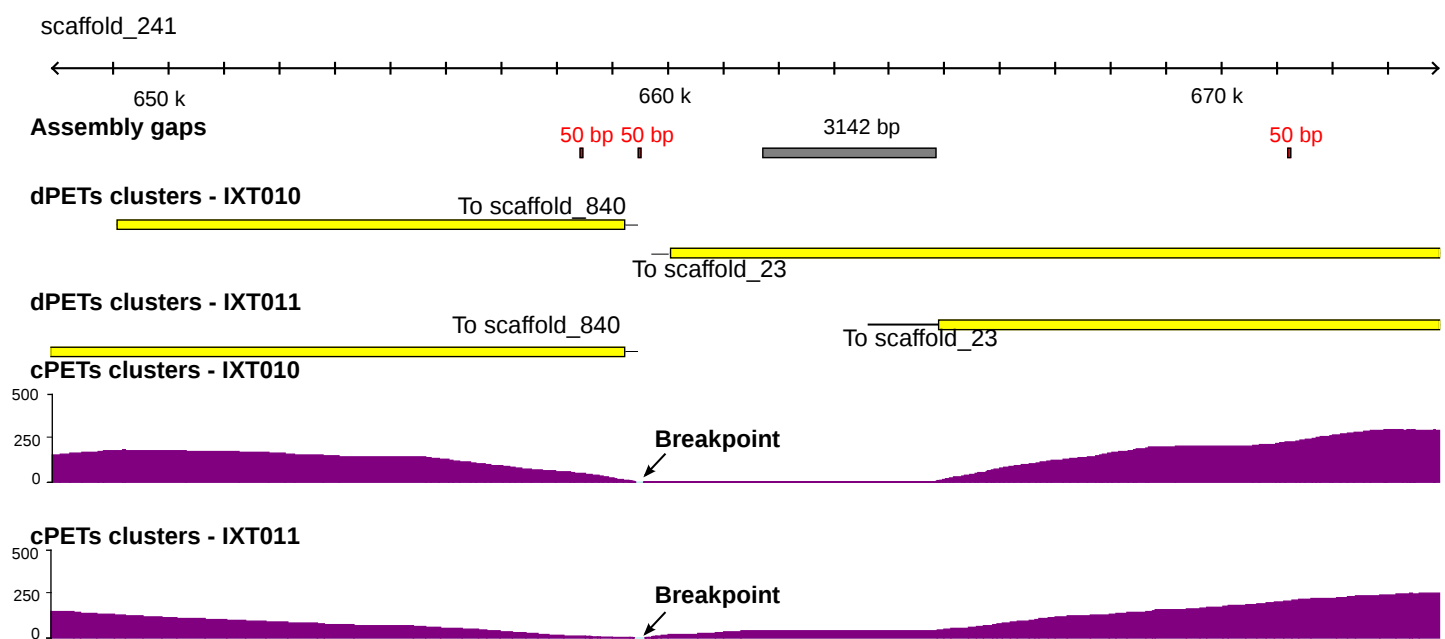

Supplement: S8 Fig — Genomic views two assembly break points. Track order: Assembly gaps, clusters of dPETs for DNA-PET libraries IXT010 and IXT011, cPETs coverage profile computed from libraries IXT010 and IXT011. The average insert size for these libraries is 9.6Kb and 17Kb, respectively. Numbers above assembly gaps indicate their length. Values in brackets correspond to their estimated length based on cPETs datas, when available. Assembly gaps of 50bp are shown in red. Assembly breakpoints potentially occur when cPET coverage drops to zero, meaning that the two sides are not connected together, at the resolution of the DNA-PET libraries. (PDF) [file pone.0137526.s008.pdf]

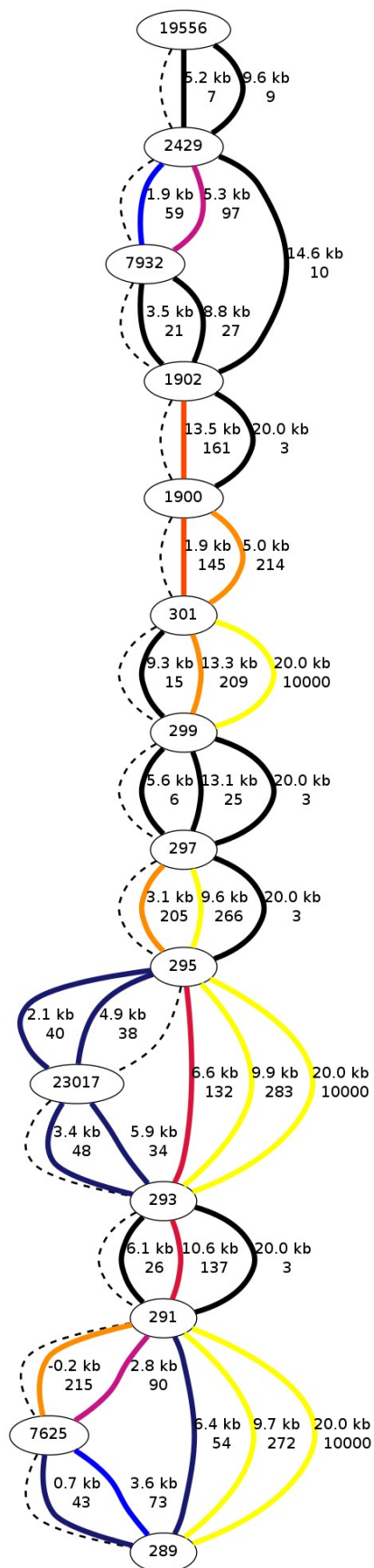

Supplement: S9 Fig — Scaffolds are shown as ovals, indicating their number (e.g. 291 for scaffold_291). The dashed line corresponds to the path through the graph and represents the final ordering of the scaffolds relative to each other. Colored lines correspond to the connection and numbers indicate the estimated distance between scaffolds and well as dPETs support. Colors are heat map coded. In this example, a few small scaffolds (scaffold_7625, scaffold_23017, scaffold_7932) are nested between longer ones. (PDF) [file pone.0137526.s009.pdf]

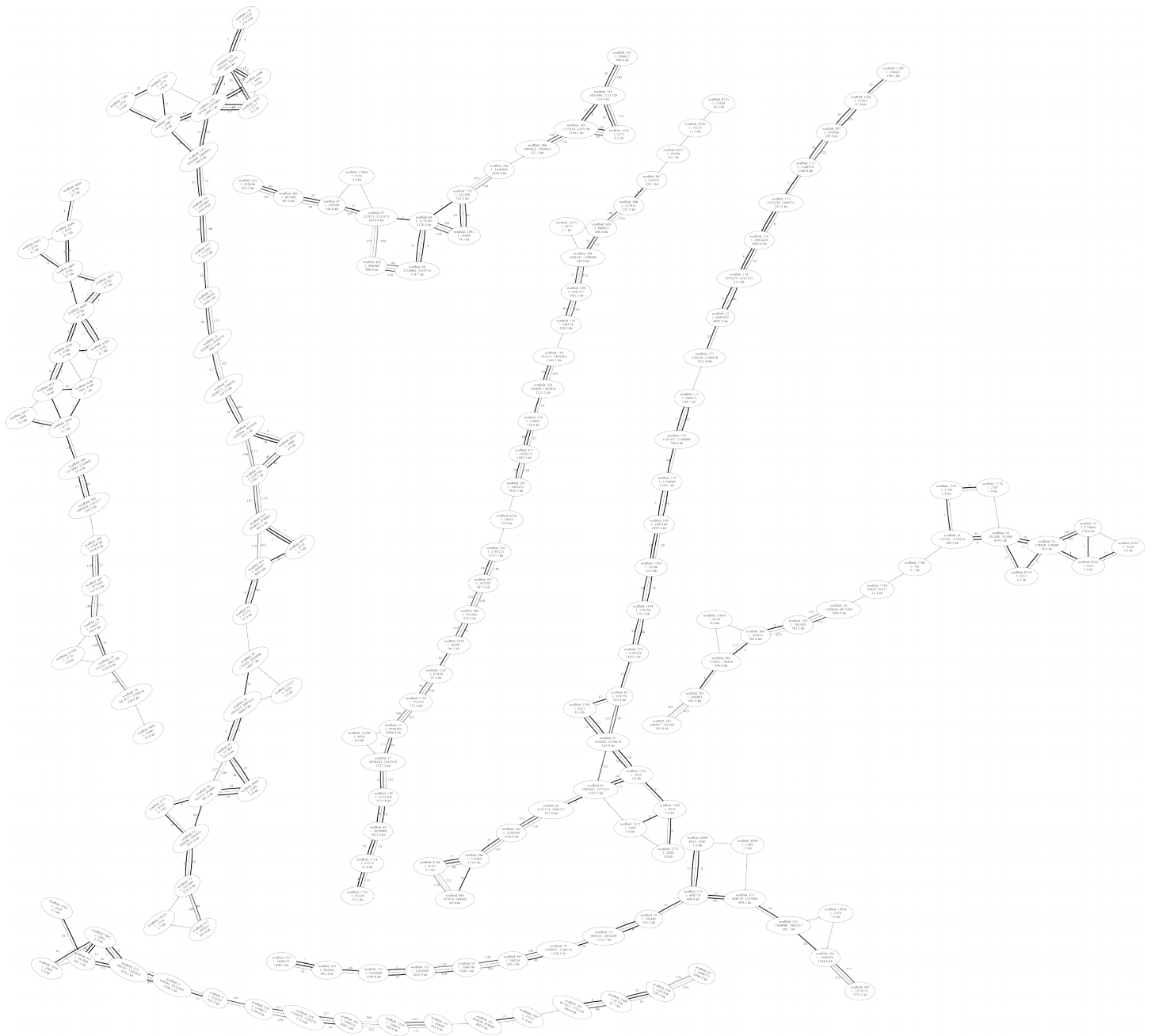

Supplement: S10 Fig — Scaffolds are shown as ovals, indicating their name. Colored lines correspond to the connection and numbers indicate the dPETs support. Colors are heat map coded. Bubble-like structure correspond to small scaffolds inserted into larger ones or result from the complex connectivity between tandemly arranged short scaffolds. (PDF) [file pone.0137526.s010.pdf]

**A**

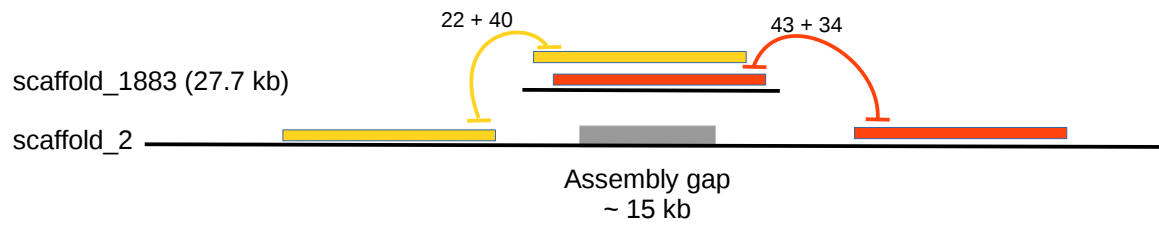

**B**

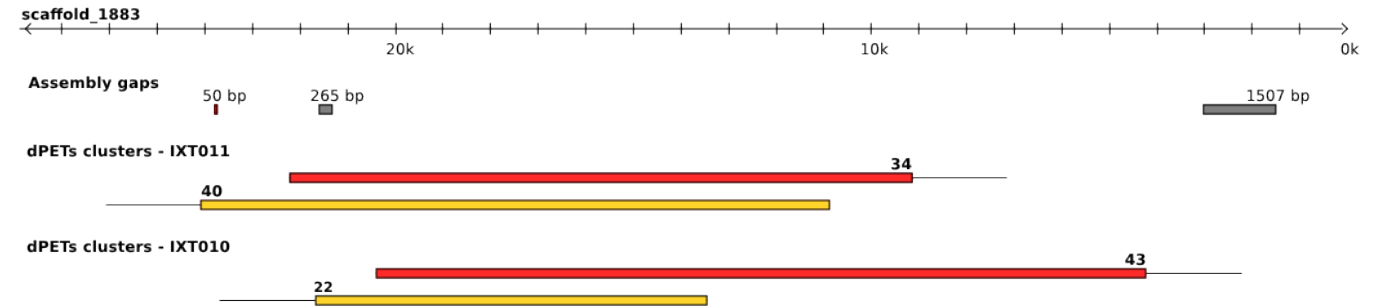

**C**

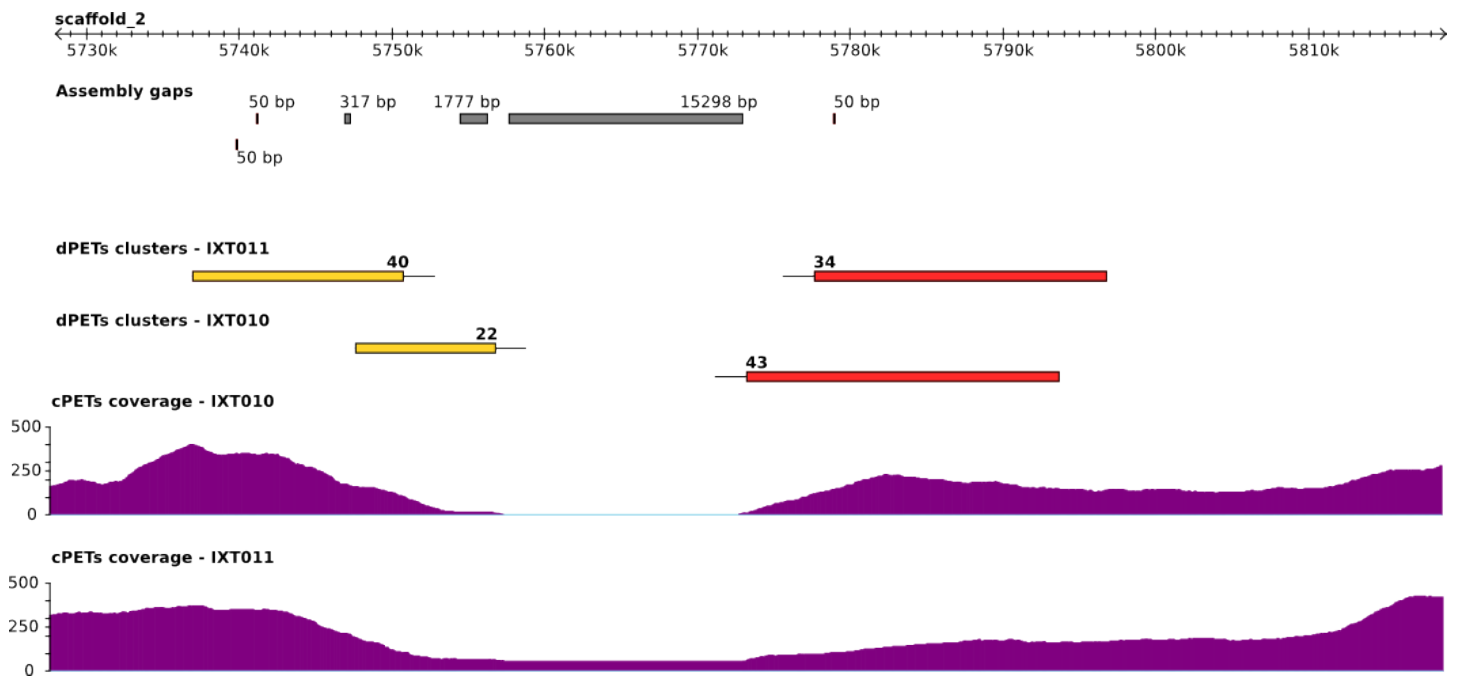

Supplement: S12 Fig — A. Conceptual representation of the connectivity between scaffolds_1883 and scaffold_2. Numbers above the links indicate the cPET count for each library. Clusters of cPETs connecting the left and the right side are drawn in yellow and orange, respectively. B, C. Detailed view of the entire scaffold_1883 and a sub-region of scaffold_2. The last two tracks correspond to the coverage density of cPET, for each DNA-PET library. (PDF) [file pone.0137526.s012.pdf]

A

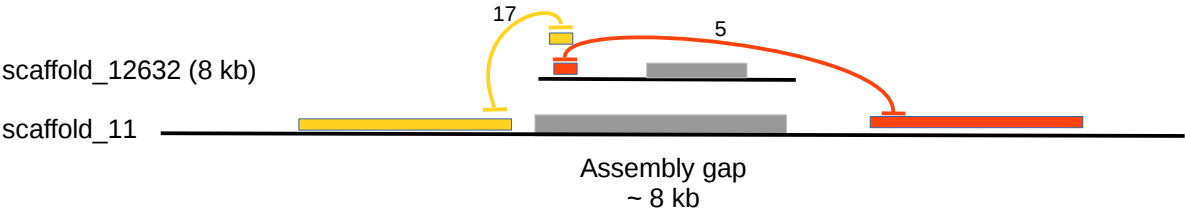

B

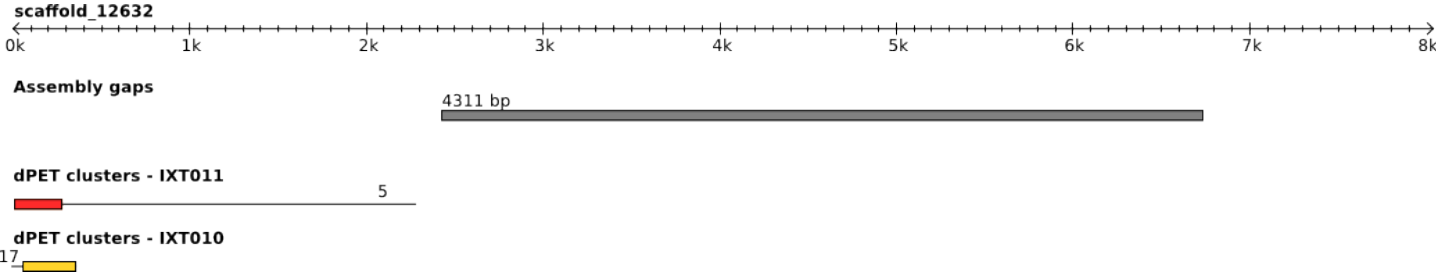

C

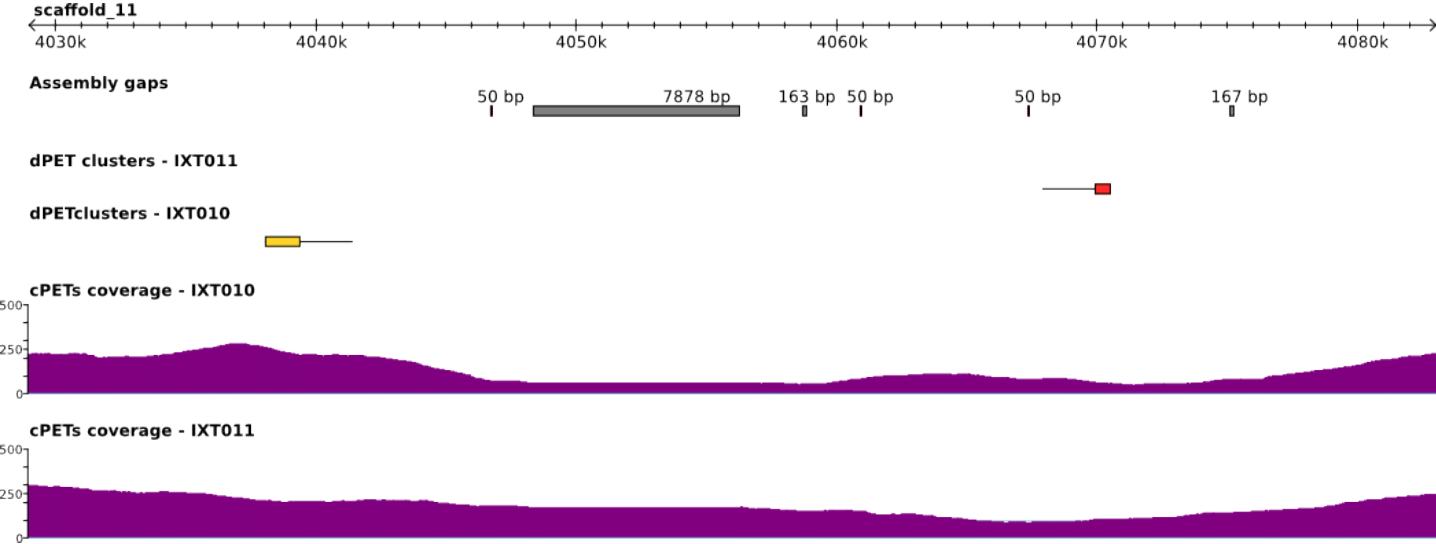

Supplement: S13 Fig — A. Conceptual representation of the connectivity between scaffolds_12632 and scaffold_11. Numbers above the links indicate the cPET count for each DNA-PET library. Clusters of cPETs connecting the left and the right side are drawn in yellow and orange, respectively. B, C. Detailed view of the entire scaffold_12632 and a subregion of scaffold_11. The last two tracks correspond to the coverage density of cPET, for each DNA-PET library. Note that the ~8Kb assembly gap on scaffold_11 is small compared to the average insert size of the two libraries. As a result, cPET coverage does not drop to zero. (PDF) [file pone.0137526.s013.pdf]

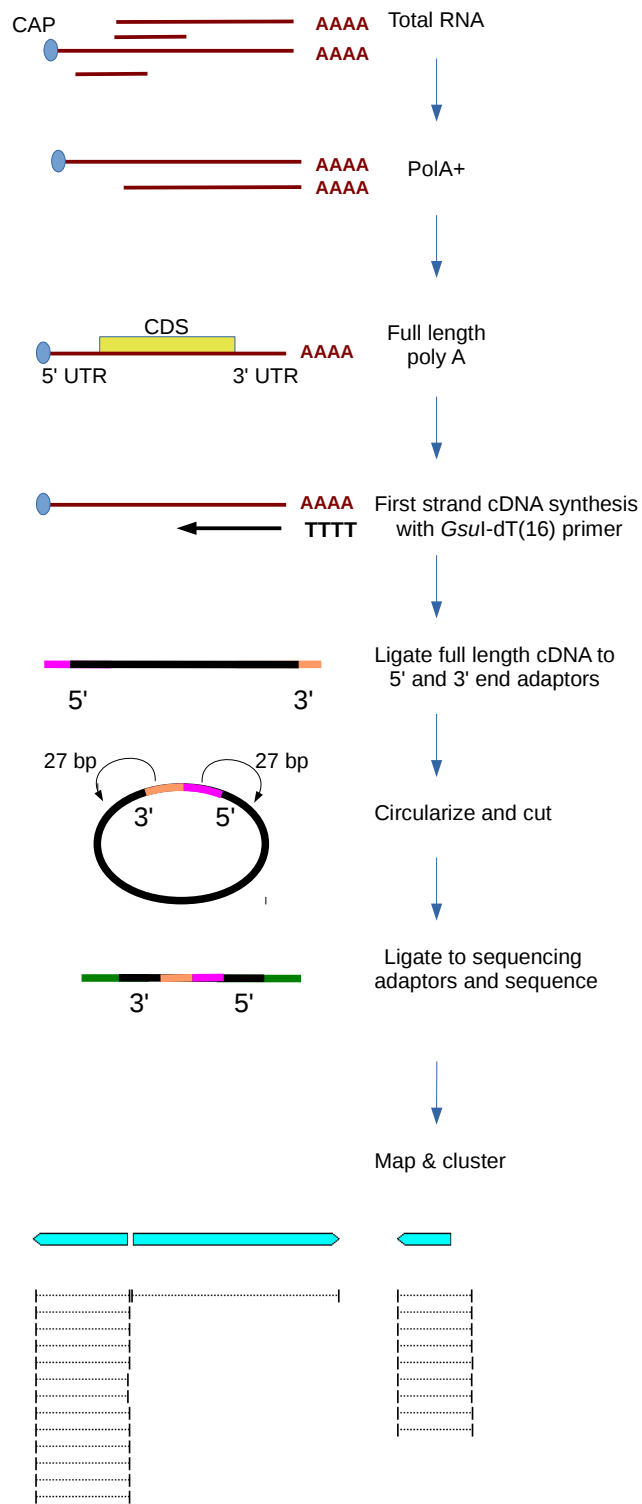

Supplement: S15 Fig — Full-length polyA+ RNA are used as template to generate full-length cDNAs. Double stranded adaptors specific for the 5' and 3' ends (pink, orange) are ligated to the ends of the transcripts. Adaptors contain an EcoP15I restriction site, cutting 27bp away from its binding site. After circularization and restriction with EcoP15I, sequencing adaptors are ligated to the resulting Paired-End diTag, prior to sequencing, mapping and clustering. (PDF) [file pone.0137526.s015.pdf]

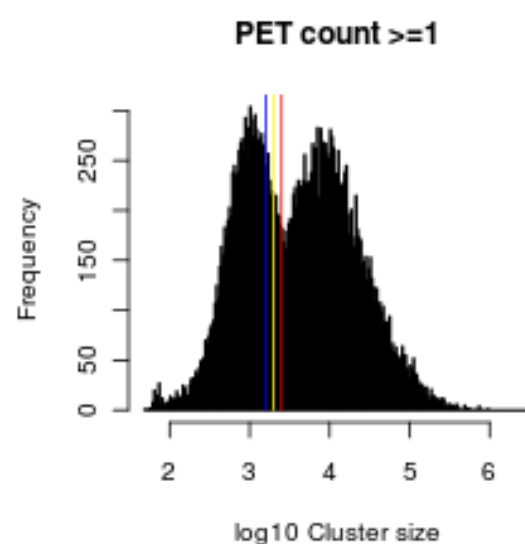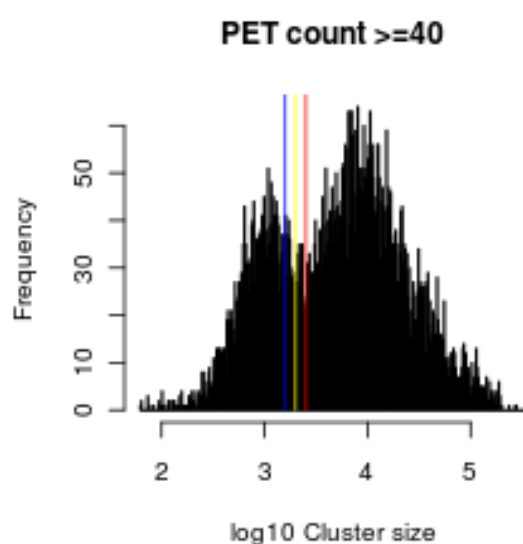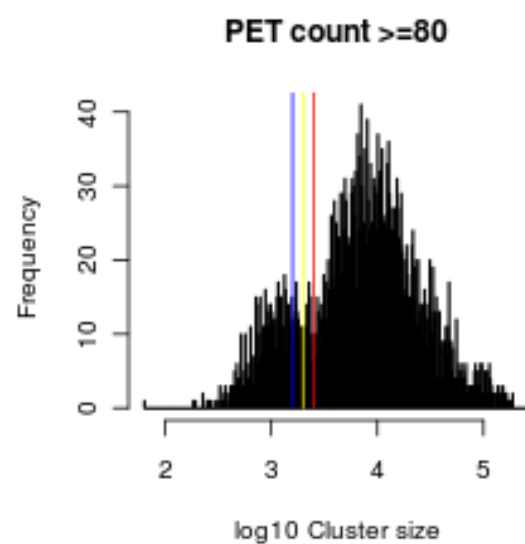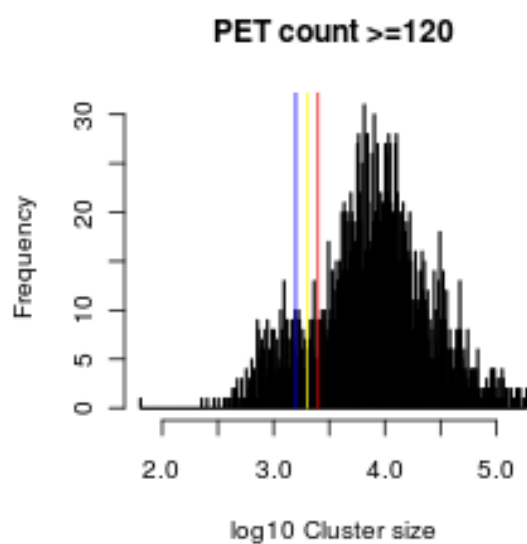

Supplement: S17 Fig — Blue, yellow and red lines denote ~1.5, ~2.0 and ~2.5Kb, respectively. (PDF) [file pone.0137526.s017.pdf]

**PET count  $\geq 1$**

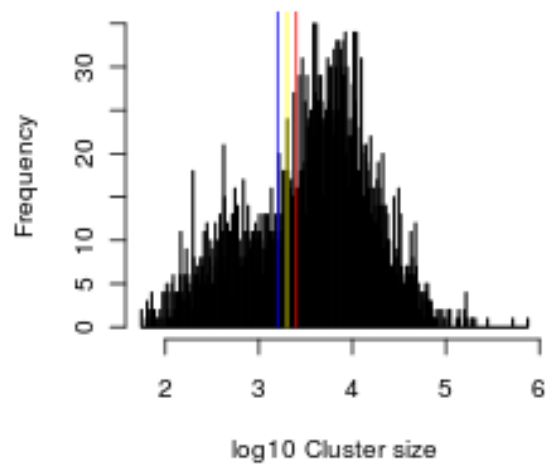

**PET count  $\geq 40$**

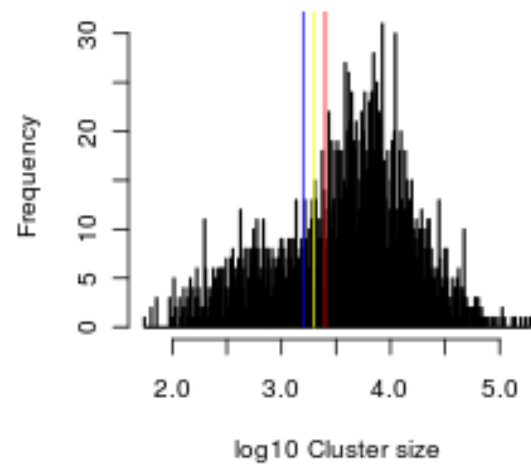

**PET count  $\geq 80$**

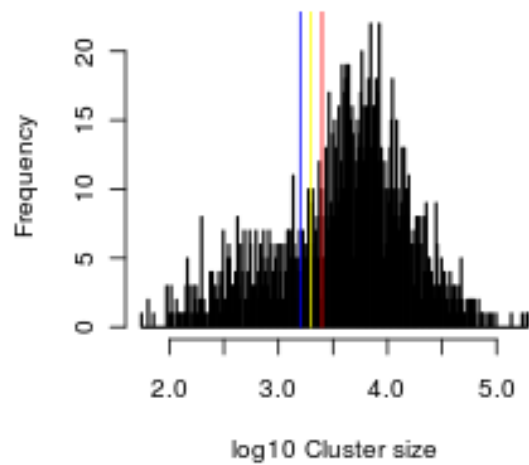

**PET count  $\geq 120$**

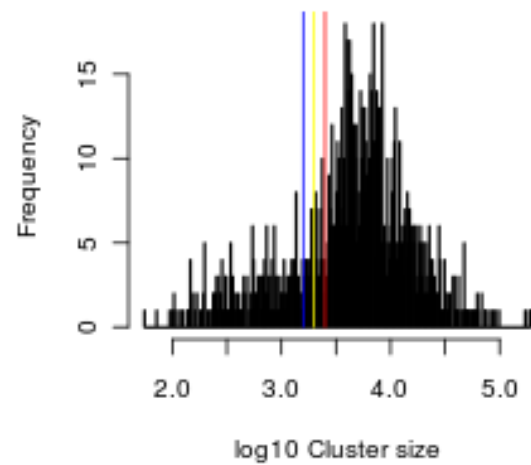

Supplement: S18 Fig — Blue, yellow and red lines denote ~1.5, ~2.0 and ~2.5Kb, respectively. (PDF) [file pone.0137526.s018.pdf]

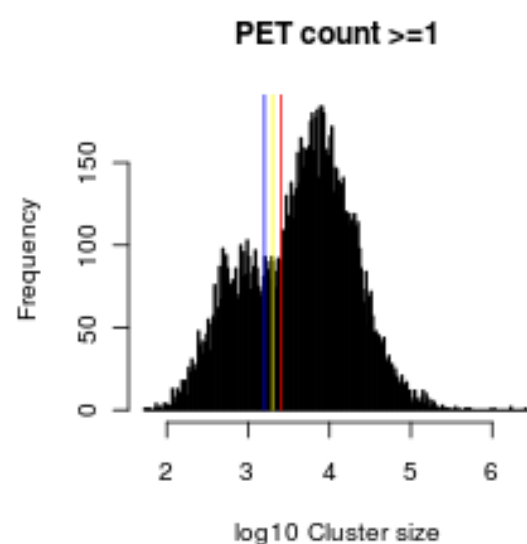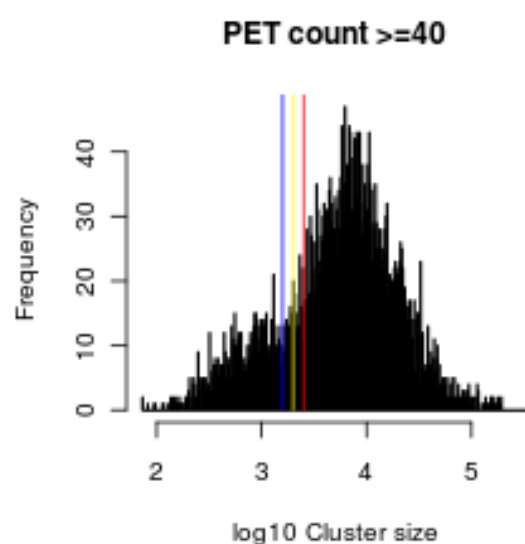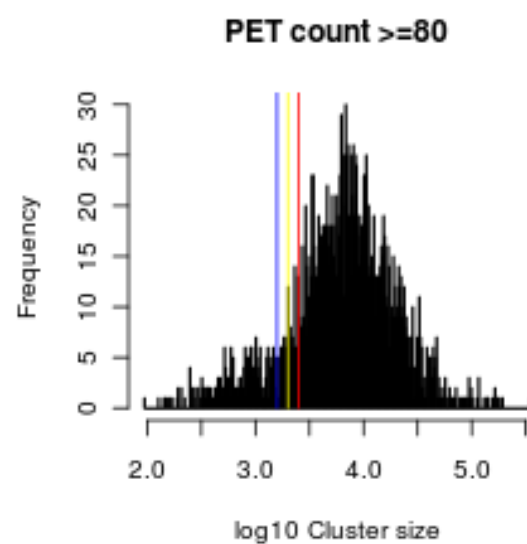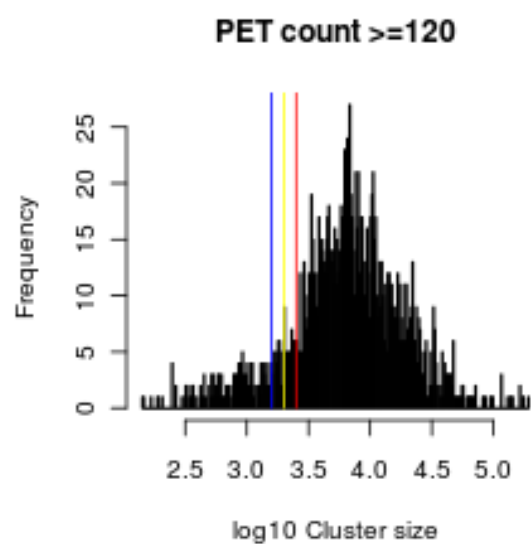

Supplement: S19 Fig — Blue, yellow and red lines denote ~1.5, ~2.0 and ~2.5Kb, respectively. (PDF) [file pone.0137526.s019.pdf]

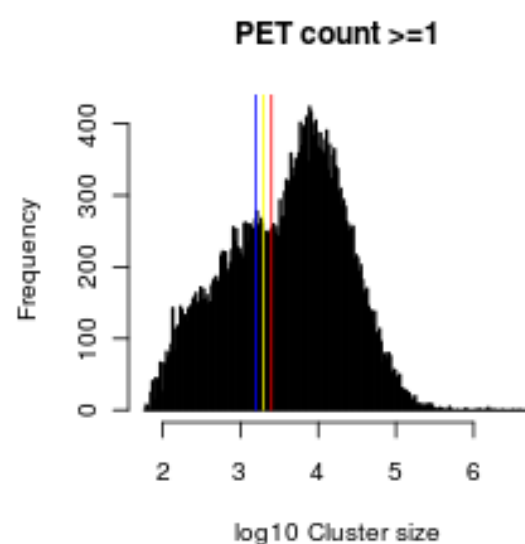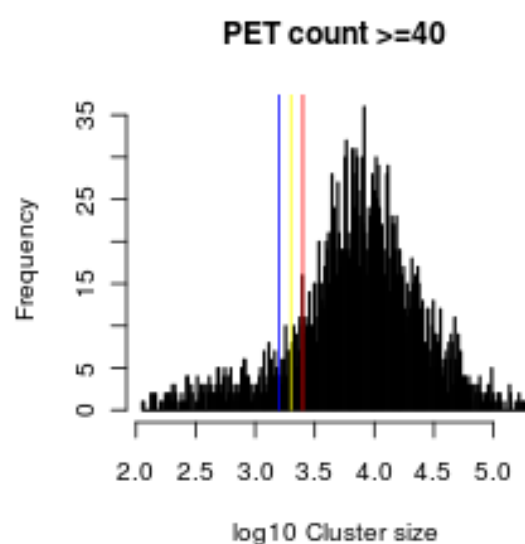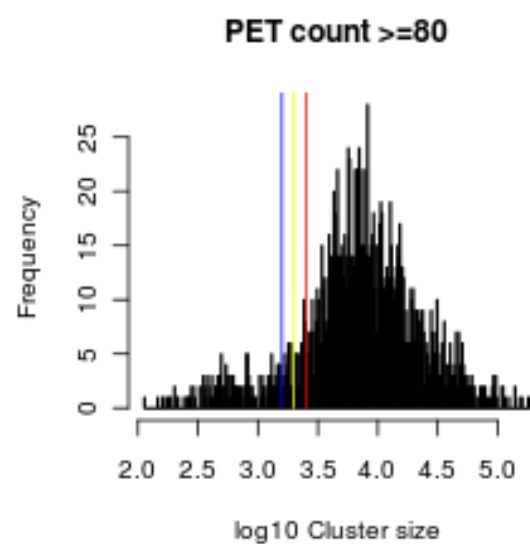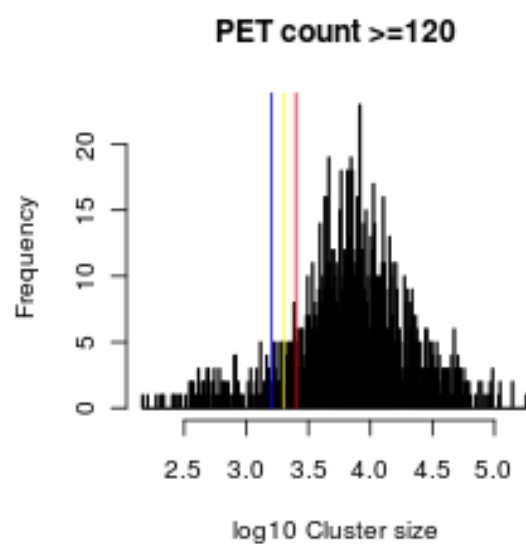

Supplement: S20 Fig — Blue, yellow and red lines denote ~1.5, ~2.0 and ~2.5Kb, respectively. (PDF) [file pone.0137526.s020.pdf]

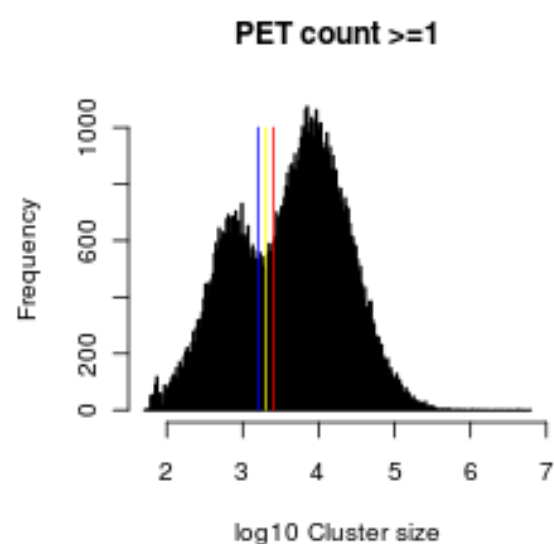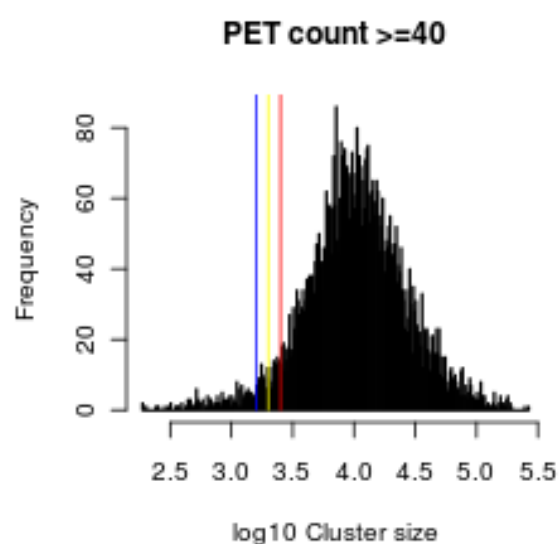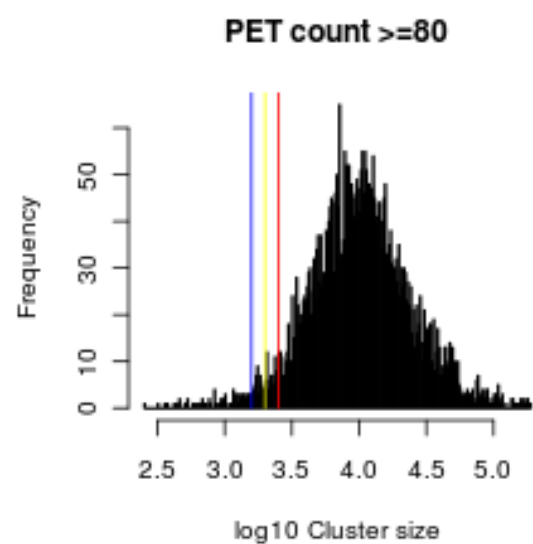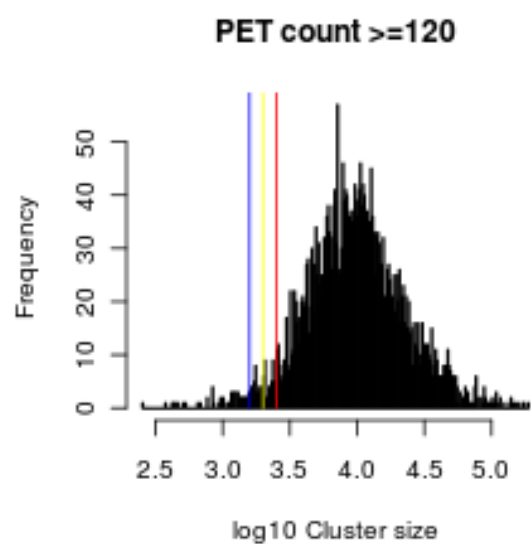

Supplement: S21 Fig — Blue, yellow and red lines denote ~1.5, ~2.0 and ~2.5Kb, respectively. (PDF) [file pone.0137526.s021.pdf]

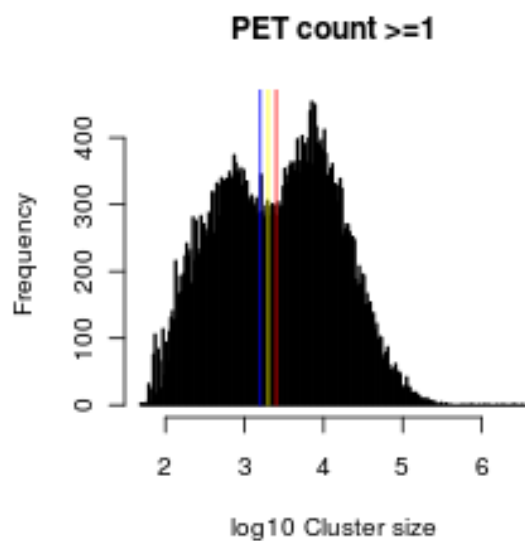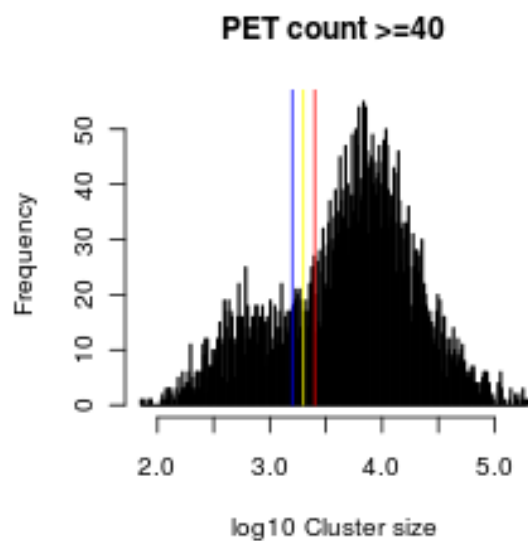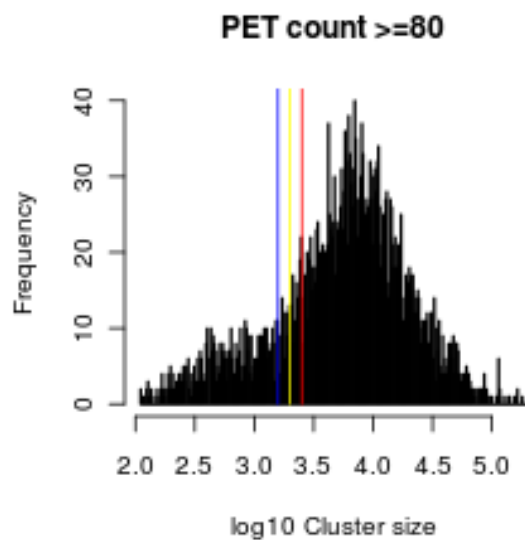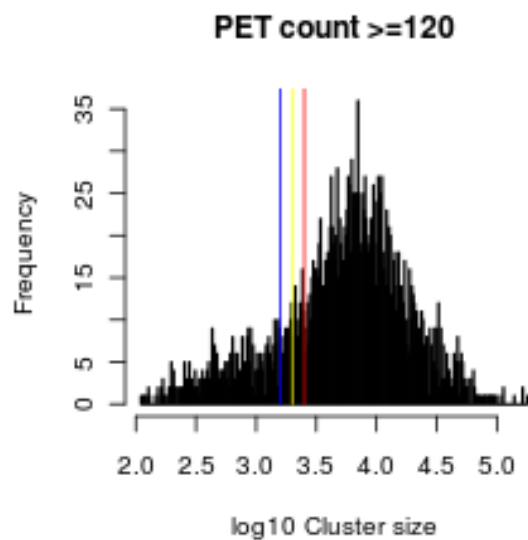

Supplement: S22 Fig — Blue, yellow and red lines denote ~1.5, ~2.0 and ~2.5Kb, respectively. (PDF) [file pone.0137526.s022.pdf]

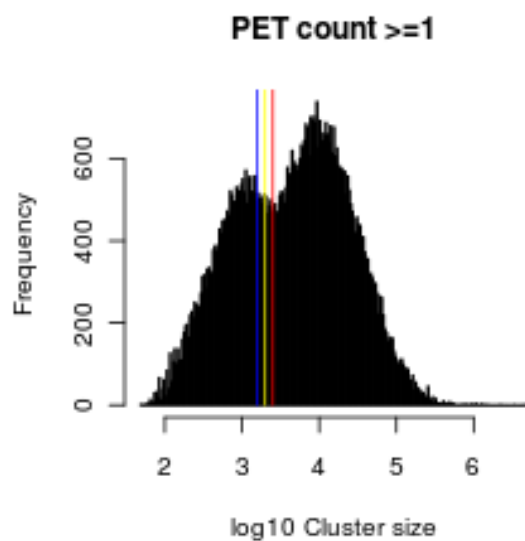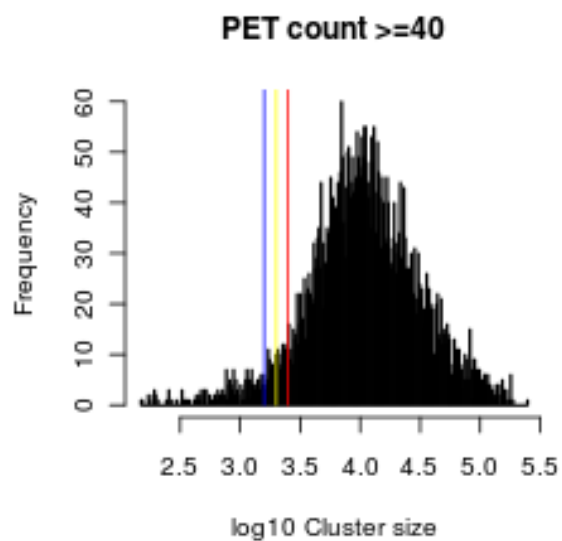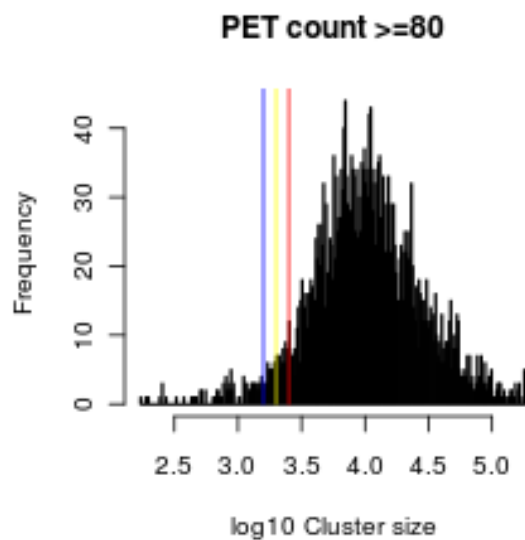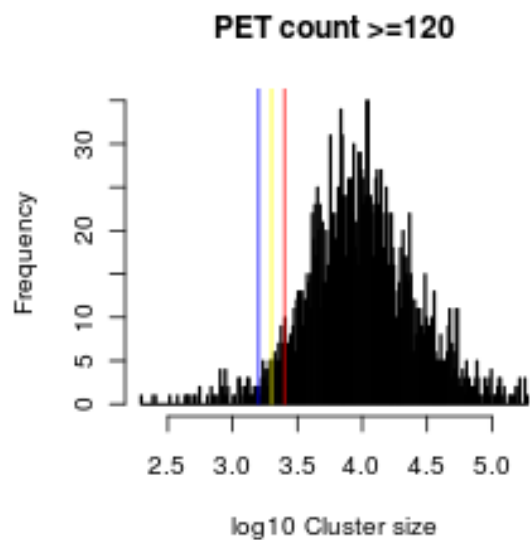

Supplement: S23 Fig — Blue, yellow and red lines denote ~1.5, ~2.0 and ~2.5Kb, respectively. (PDF) [file pone.0137526.s023.pdf]

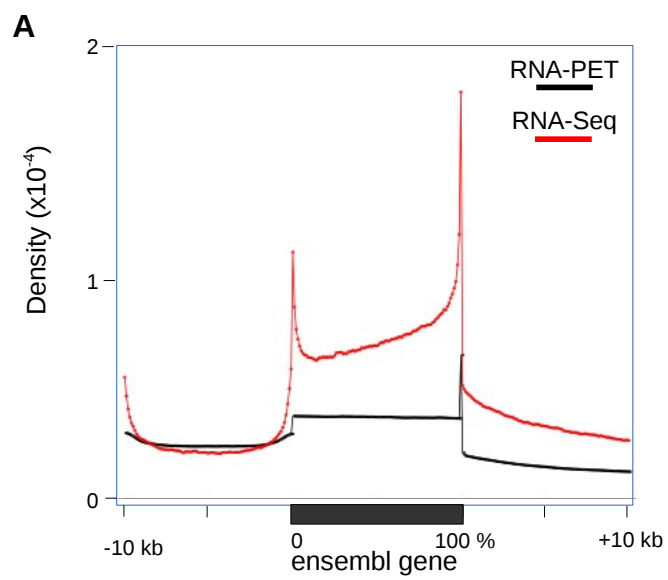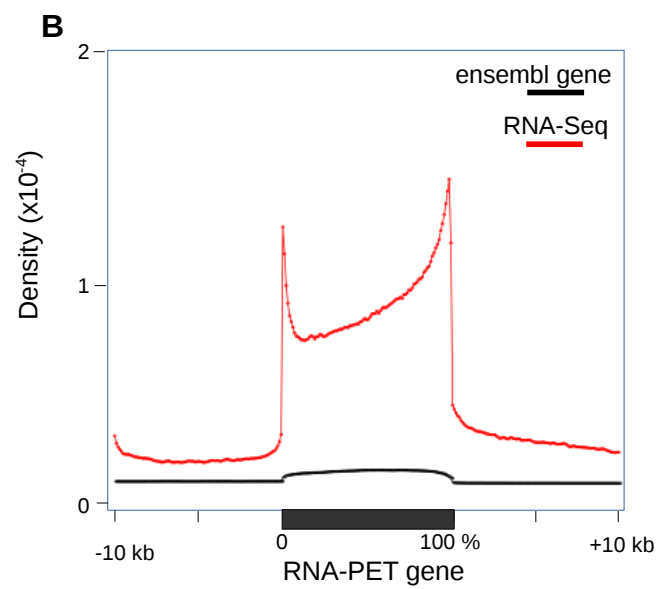

Supplement: S24 Fig — A. Density distribution of RNA-Seq reads and RNA-PET ditags over Ensembl gene models. B. Density distribution of RNA-Seq reads and Ensembl genes boundaries over RNA-PET based gene models. Gene coverage is expressed in percent of gene length. (PDF) [file pone.0137526.s024.pdf]

### Gene models size

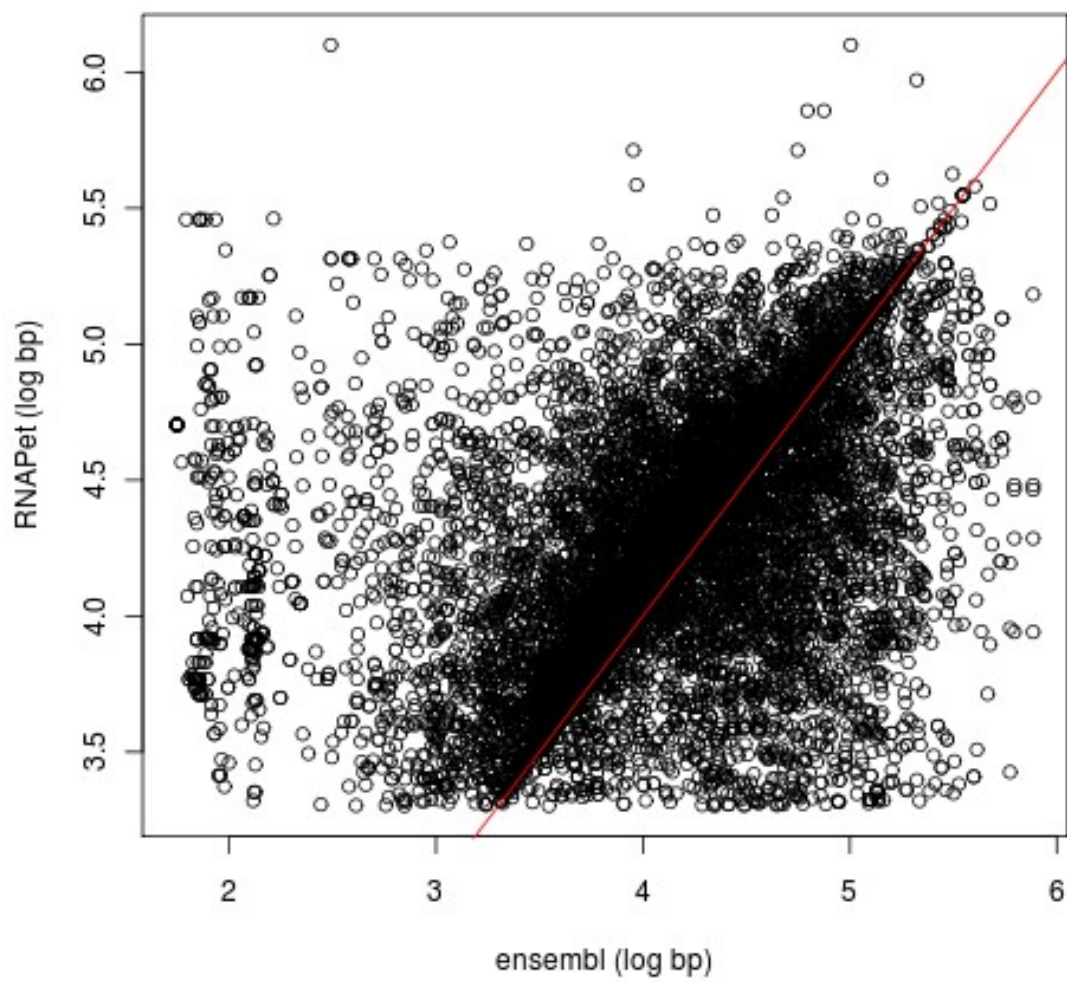

Supplement: S25 Fig — The plot corresponds to the size of individual RNA-PET-based gene models relative to their Ensembl counterpart. Gene models of the same size between the two datasets would follow the red line. (PDF) [file pone.0137526.s025.pdf]

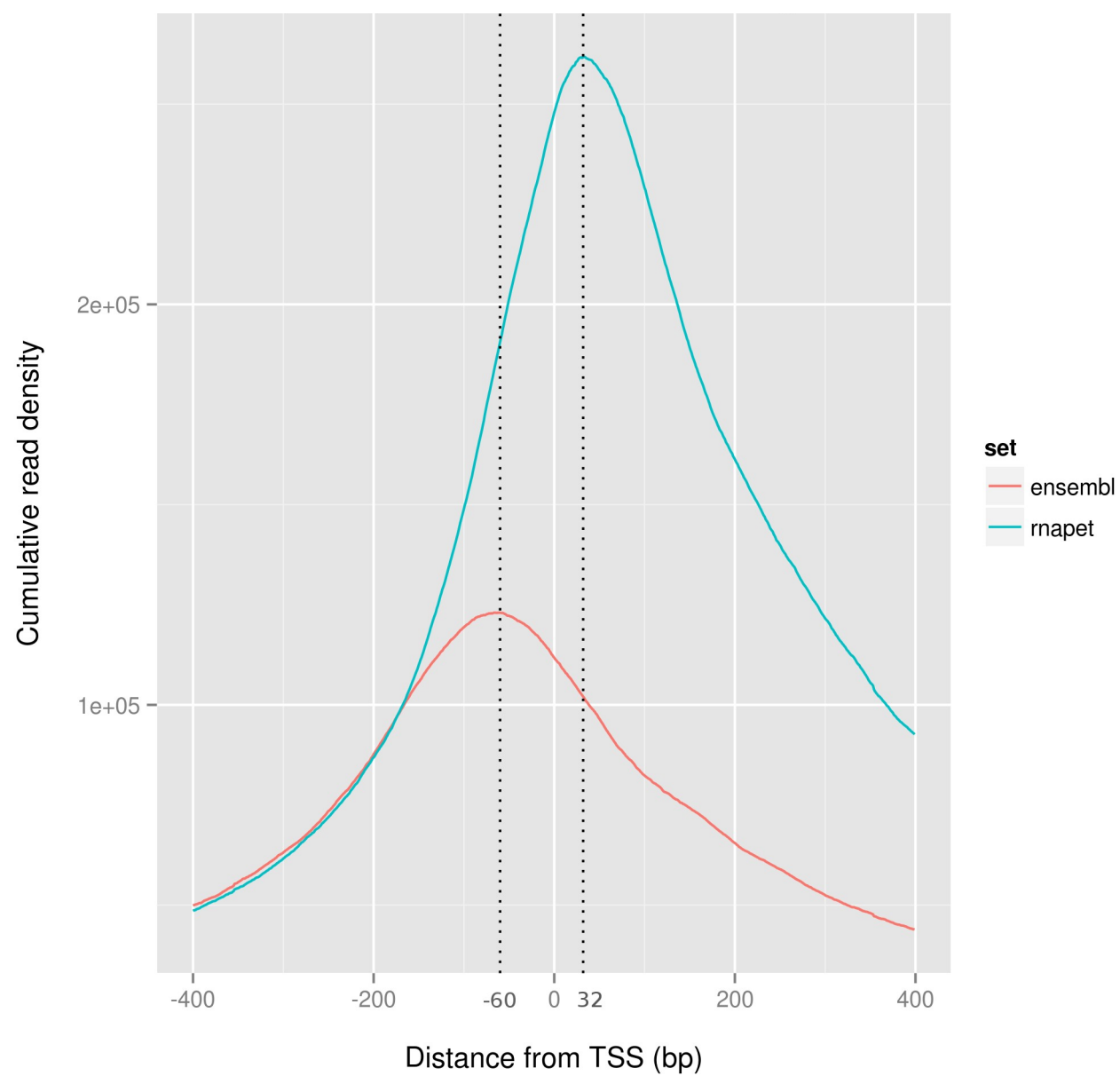

Supplement: S26 Fig — The RNA-Pol II density was computed at the 5' end of gene models, based on Ensembl and RNA-PET. The 5' end of Ensembl models is located 60bp downstream of the RNA-Pol II peak (red curve), typically located ~25–45pb downstream the TSS. In contrast, the 5' end of models enriched with RNA-PET data extend 32bp upstream of RNA-Pol II peak. (PDF) [file pone.0137526.s026.pdf]
