# Supplementary material for: Xenopus tropicalis Genome Re-Scaffolding and Re-Annotation Reach the Resolution Required for In Vivo ChIA-PET Analysis
Source: PLoS One. 2015 Sep 8;10(9):e0137526. doi: 10.1371/journal.pone.0137526 (PMC4562602; doi:10.1371/journal.pone.0137526)
Supplement: S4 Fig — Individual cPETs spanning a single assembly gap of 50bp have been used to estimate their actual length. For each cPET, the measured span size (left panel) was compared to the expected span size. Disagreements between expected and measured span sizes were used to derive independent estimate of the gap actual size (right panel). The number of cPETs, the average and median of the estimates are shown. A, B, C are three illustrative examples. (PDF) [file pone.0137526.s004.pdf]

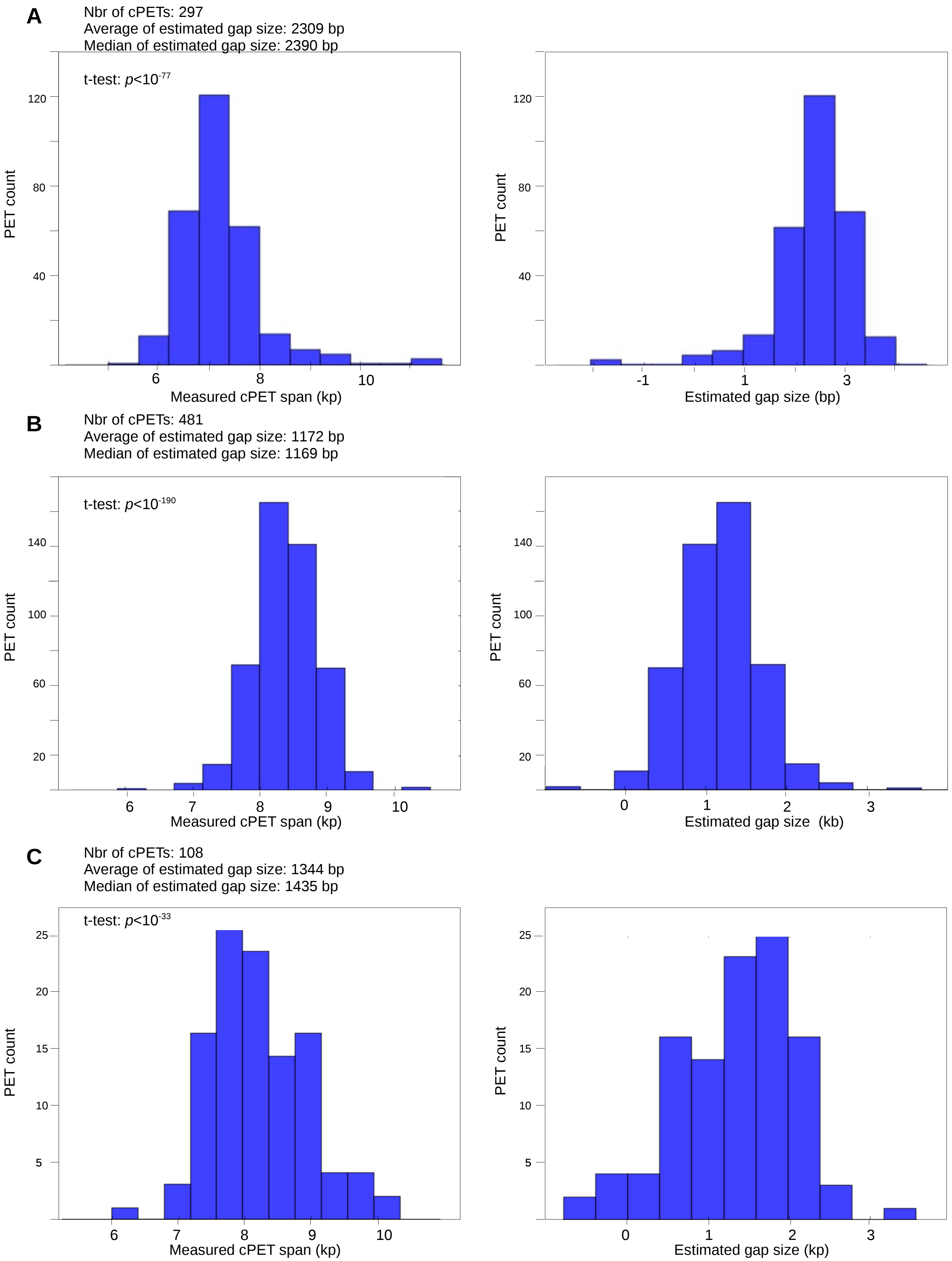
A
Nbr of cPETs: 297
Average of estimated gap size: 2309 bp
Median of estimated gap size: 2390 bp
t-test: p<10-77
PET count
120
80
40
6
8
10
Measured cPET span (kp)
PET count
120
80
40
-1
1
3
Estimated gap size (bp)
B
Nbr of cPETs: 481
Average of estimated gap size: 1172 bp
Median of estimated gap size: 1169 bp
t-test: p<10-190
PET count
140
100
60
20
6
7
8
9
10
Measured cPET span (kp)
PET count
140
100
60
20
0
1
2
3
Estimated gap size (kb)
C
Nbr of cPETs: 108
Average of estimated gap size: 1344 bp
Median of estimated gap size: 1435 bp
t-test: p<10-33
PET count
25
20
15
10
5
6
7
8
9
10
Measured cPET span (kp)
PET count
25
20
15
10
5
0
1
2
3
Estimated gap size (kp)
