# Supplementary material for: Xenopus tropicalis Genome Re-Scaffolding and Re-Annotation Reach the Resolution Required for In Vivo ChIA-PET Analysis
Source: PLoS One. 2015 Sep 8;10(9):e0137526. doi: 10.1371/journal.pone.0137526 (PMC4562602; doi:10.1371/journal.pone.0137526)
Supplement: S5 Fig — In a number of cases, the measure of the actual gaps length follows a bimodal distribution, with two populations labeled α and β. See legend of S4 Fig for details. A, B and C are illustrative examples. (PDF) [file pone.0137526.s005.pdf]

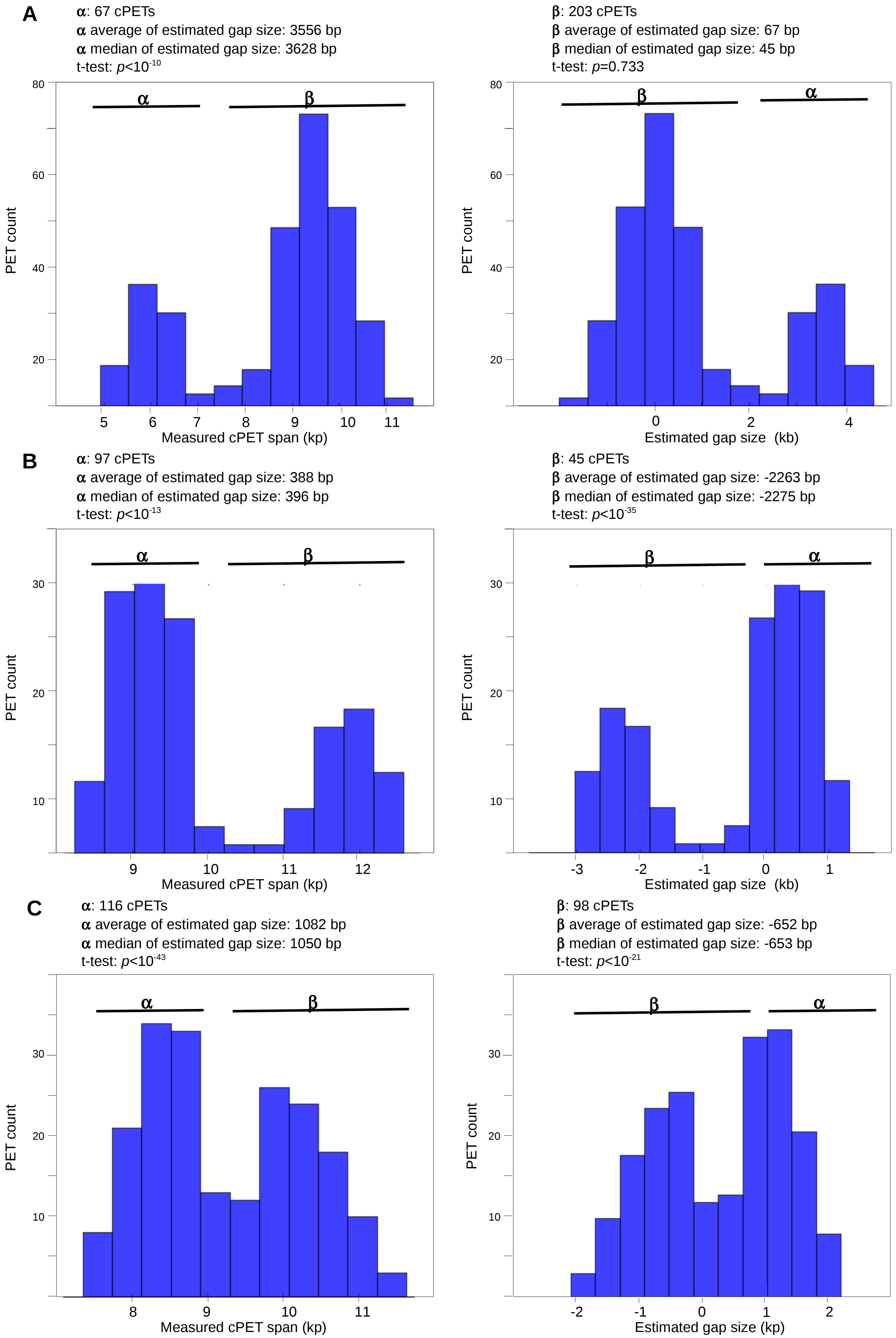
A
α: 67 cPETs
α average of estimated gap size: 3556 bp
α median of estimated gap size: 3628 bp
t-test: p<10-10
β: 203 cPETs
β average of estimated gap size: 67 bp
β median of estimated gap size: 45 bp
t-test: p=0.733
PET count
Measured cPET span (kp)
Estimated gap size (kb)
B
α: 97 cPETs
α average of estimated gap size: 388 bp
α median of estimated gap size: 396 bp
t-test: p<10-13
β: 45 cPETs
β average of estimated gap size: -2263 bp
β median of estimated gap size: -2275 bp
t-test: p<10-35
PET count
Measured cPET span (kp)
Estimated gap size (kb)
C
α: 116 cPETs
α average of estimated gap size: 1082 bp
α median of estimated gap size: 1050 bp
t-test: p<10-43
β: 98 cPETs
β average of estimated gap size: -652 bp
β median of estimated gap size: -653 bp
t-test: p<10-21
PET count
Measured cPET span (kp)
Estimated gap size (kp)
