# Supplementary material for: Xenopus tropicalis Genome Re-Scaffolding and Re-Annotation Reach the Resolution Required for In Vivo ChIA-PET Analysis
Source: PLoS One. 2015 Sep 8;10(9):e0137526. doi: 10.1371/journal.pone.0137526 (PMC4562602; doi:10.1371/journal.pone.0137526)
Supplement: S6 Fig — Individual cPETs spanning a single assembly gap (≠ 50bp) have been used to estimate their actual size. See legend of S4 Fig for details. The number of cPETs, the expected size, the average and median of the estimates are shown. A, B, C are three illustrative examples. (PDF) [file pone.0137526.s006.pdf]

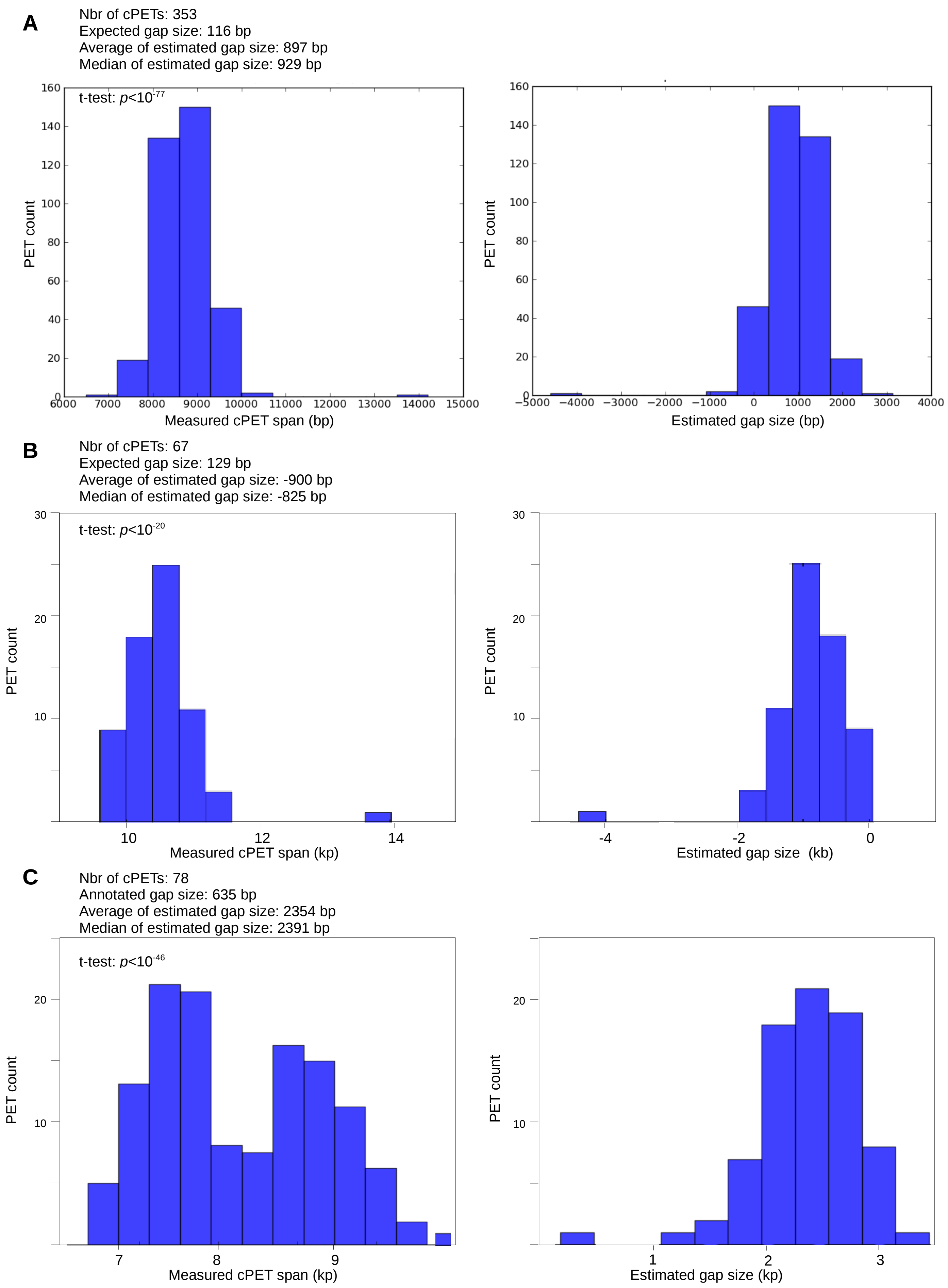
A
Nbr of cPETs: 353
Expected gap size: 116 bp
Average of estimated gap size: 897 bp
Median of estimated gap size: 929 bp
t-test: $p<10^{-77}$
PET count
Measured cPET span (bp)
Estimated gap size (bp)
B
Nbr of cPETs: 67
Expected gap size: 129 bp
Average of estimated gap size: -900 bp
Median of estimated gap size: -825 bp
t-test: $p<10^{-20}$
PET count
Measured cPET span (kp)
Estimated gap size (kb)
C
Nbr of cPETs: 78
Annotated gap size: 635 bp
Average of estimated gap size: 2354 bp
Median of estimated gap size: 2391 bp
t-test: $p<10^{-46}$
PET count
Measured cPET span (kp)
Estimated gap size (kp)
