# Supplementary material for: Xenopus tropicalis Genome Re-Scaffolding and Re-Annotation Reach the Resolution Required for In Vivo ChIA-PET Analysis
Source: PLoS One. 2015 Sep 8;10(9):e0137526. doi: 10.1371/journal.pone.0137526 (PMC4562602; doi:10.1371/journal.pone.0137526)
Supplement: S11 Fig — Scaffolds' fragments are shown as ovals, indicating their name (e.g. 291 for scaffold_291), which part of the original scaffold they correspond to, and their size. The dashed line corresponds to the path through the graph and represents the final ordering of the scaffolds relative to each other. Colored lines correspond to the connection and numbers indicate the dPETs support. Colors are heat map coded. A and B represent two examples of small scaffolds being inserted into assembly gaps present in larger ones. (PDF) [file pone.0137526.s011.pdf]

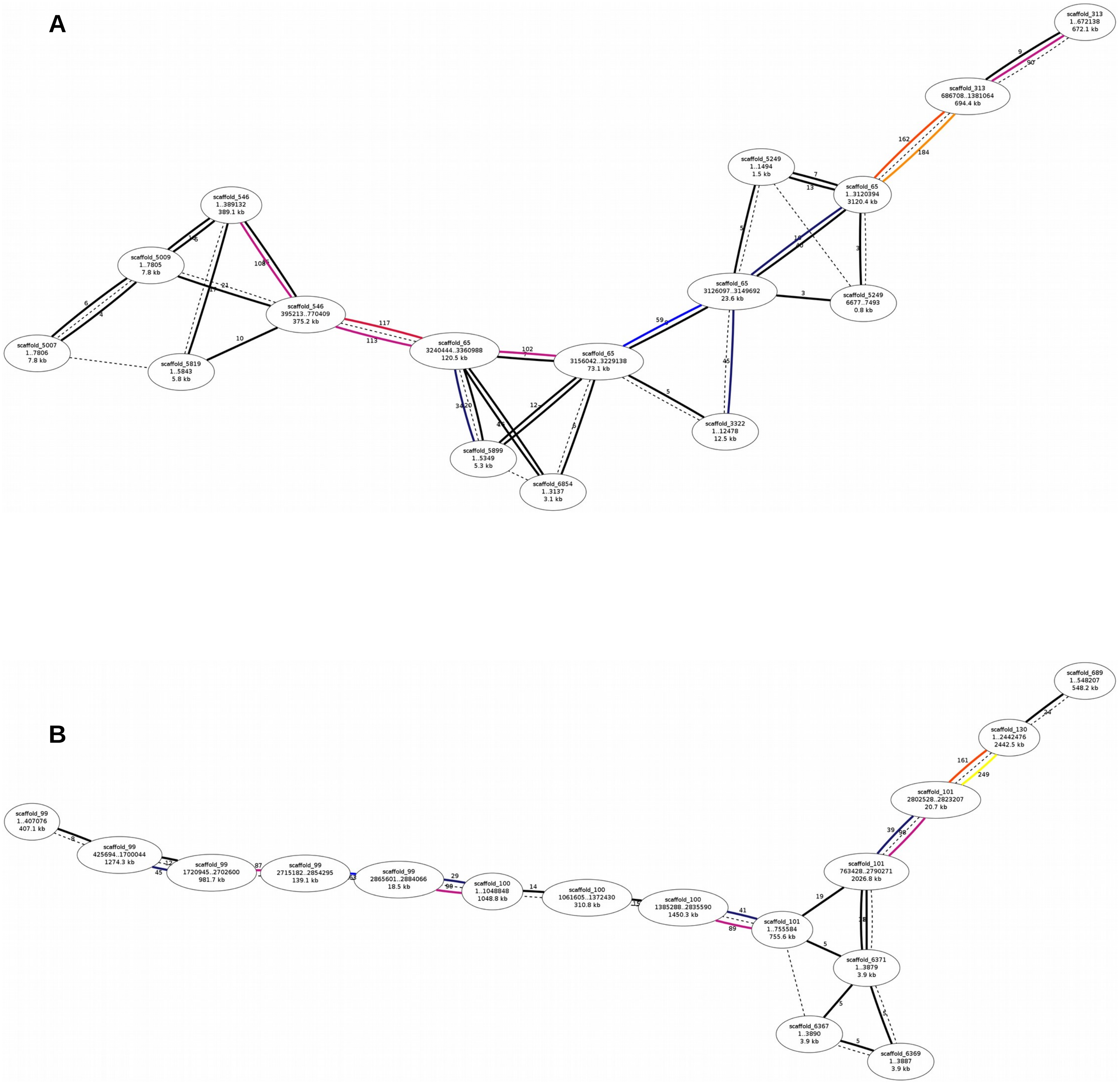
A
scaffold_313 1..672138 672.1 kb
scaffold_313 686708..1381064 694.4 kb
scaffold_5249 1..1494 1.5 kb
scaffold_65 1..3120394 3120.4 kb
scaffold_546 1..389132 389.1 kb
scaffold_5009 1..7805 7.8 kb
scaffold_65 3126097..3149692 23.6 kb
scaffold_5249 6677..7493 0.8 kb
scaffold_546 395213..770409 375.2 kb
scaffold_5007 1..7806 7.8 kb
scaffold_65 3240444..3360988 120.5 kb
scaffold_65 3156042..3229138 73.1 kb
scaffold_5819 1..5843 5.8 kb
scaffold_3322 1..12478 12.5 kb
scaffold_5899 1..5349 5.3 kb
scaffold_6854 1..3137 3.1 kb
B
scaffold_689 1..548207 548.2 kb
scaffold_130 1..2442476 2442.5 kb
scaffold_101 2802528..2823207 20.7 kb
scaffold_99 1..407076 407.1 kb
scaffold_99 425694..1700044 1274.3 kb
scaffold_99 1720945..2702600 981.7 kb
scaffold_99 2715182..2854295 139.1 kb
scaffold_99 2865601..2884066 18.5 kb
scaffold_100 1..1048848 1048.8 kb
scaffold_100 1061605..1372430 310.8 kb
scaffold_100 1385288..2835590 1450.3 kb
scaffold_101 763428..2790271 2026.8 kb
scaffold_101 1..755584 755.6 kb
scaffold_6371 1..3879 3.9 kb
scaffold_6367 1..3890 3.9 kb
scaffold_6369 1..3887 3.9 kb
