# Supplementary material for: Xenopus tropicalis Genome Re-Scaffolding and Re-Annotation Reach the Resolution Required for In Vivo ChIA-PET Analysis
Source: PLoS One. 2015 Sep 8;10(9):e0137526. doi: 10.1371/journal.pone.0137526 (PMC4562602; doi:10.1371/journal.pone.0137526)
Supplement: S14 Fig — A. Conceptual representation of the connectivity between scaffolds. Numbers above the links indicate the cPET count for each DNA-PET library. Clusters of cPETs connecting the left and the right side of the nested scaffolds are drawn in yellow/blue and orange/green. B, C, D. Detailed view of the nested scaffolds and a sub-region of scaffold_1. The last two tracks correspond to the coverage density of cPET, for each DNA-PET library. Note that the ~8Kb assembly gap on scaffold_1 is small compared to the average insert size of the two libraries. As a result, cPET coverage does not drop to zero. (PDF) [file pone.0137526.s014.pdf]

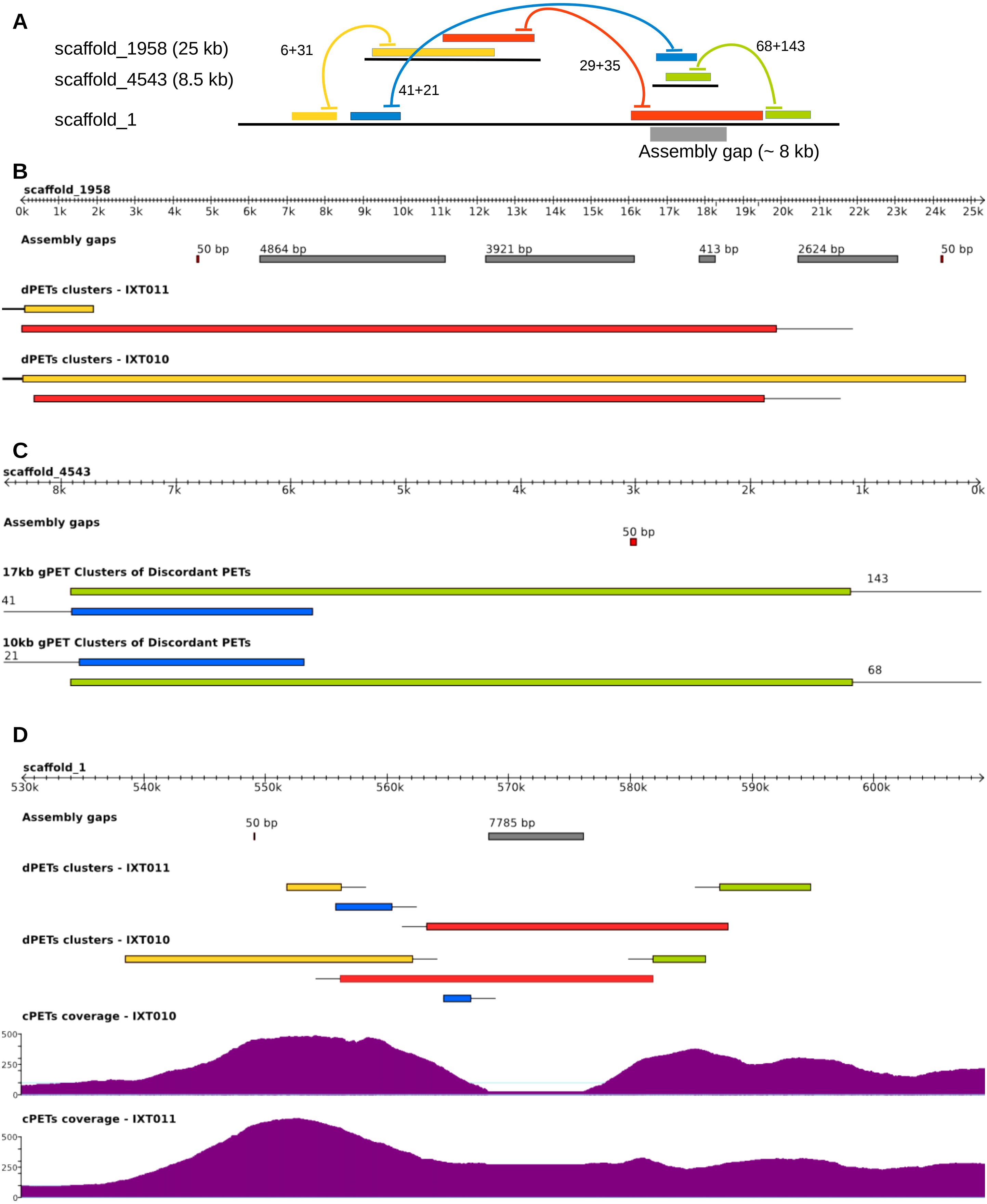
A
scaffold_1958 (25 kb)
scaffold_4543 (8.5 kb)
scaffold_1
6+31
41+21
29+35
68+143
Assembly gap (~ 8 kb)
B
scaffold_1958
0k 1k 2k 3k 4k 5k 6k 7k 8k 9k 10k 11k 12k 13k 14k 15k 16k 17k 18k 19k 20k 21k 22k 23k 24k 25k
Assembly gaps
50 bp
4864 bp
3921 bp
413 bp
2624 bp
50 bp
dPETs clusters - IXT011
dPETs clusters - IXT010
C
scaffold_4543
8k 7k 6k 5k 4k 3k 2k 1k 0k
Assembly gaps
50 bp
17kb gPET Clusters of Discordant PETs
143
41
10kb gPET Clusters of Discordant PETs
21
68
D
scaffold_1
530k 540k 550k 560k 570k 580k 590k 600k
Assembly gaps
50 bp
7785 bp
dPETs clusters - IXT011
dPETs clusters - IXT010
cPETs coverage - IXT010
500
250
0
cPETs coverage - IXT011
500
250
0
