# Supplementary material for: Xenopus tropicalis Genome Re-Scaffolding and Re-Annotation Reach the Resolution Required for In Vivo ChIA-PET Analysis
Source: PLoS One. 2015 Sep 8;10(9):e0137526. doi: 10.1371/journal.pone.0137526 (PMC4562602; doi:10.1371/journal.pone.0137526)
Supplement: S16 Fig — The adaptor at the 3' end of transcripts is labeled with a specific signature (AACTGCTG). RNA-PET di-tags are labeled HT (Head and Tail), TT (Tail Tail) or HH (Head Head) on whether the signature was found at one end, both or none, respectively. Venn diagrams represent the proportion of HT, HH and TT di-tags obtained for each tissue library. Numbers indicate the total number of PETs per category. (PDF) [file pone.0137526.s016.pdf]

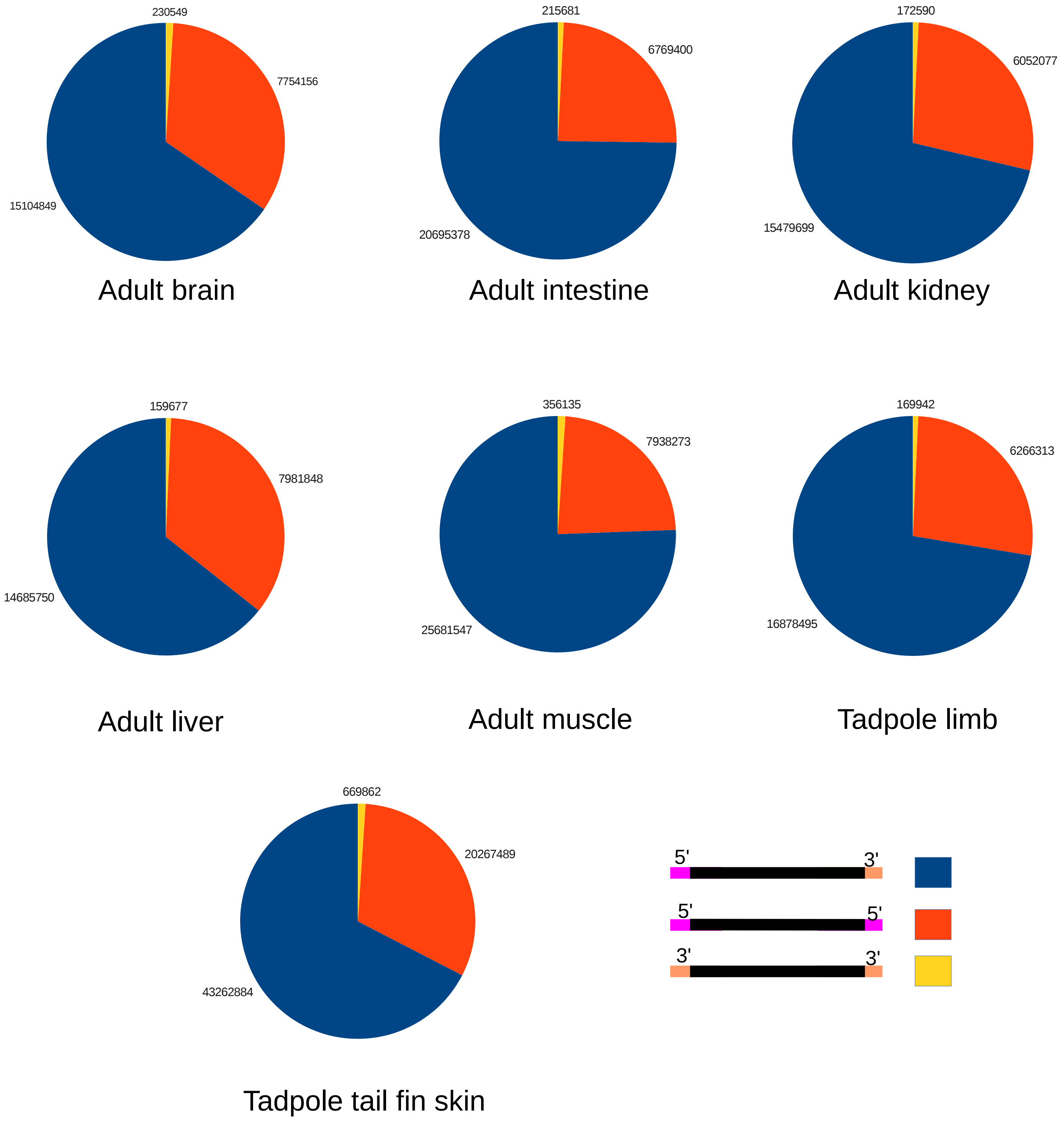
230549
7754156
15104849
Adult brain
215681
6769400
20695378
Adult intestine
172590
6052077
15479699
Adult kidney
159677
7981848
14685750
Adult liver
356135
7938273
25681547
Adult muscle
169942
6266313
16878495
Tadpole limb
669862
20267489
43262884
Tadpole tail fin skin
5'
3'
5'
5'
3'
3'
